# Determinants of the Changes in Glycemic Control with Exercise Training in Type 2 Diabetes: A Randomized Trial

**DOI:** 10.1371/journal.pone.0062973

**Published:** 2013-06-21

**Authors:** Neil M. Johannsen, Lauren M. Sparks, Zhengyu Zhang, Conrad P. Earnest, Steven R. Smith, Timothy S. Church, Eric Ravussin

**Affiliations:** 1 Pennington Biomedical Research Center, Baton Rouge, Louisiana, United States of America; 2 Louisiana State University, Baton Rouge, Louisiana, United States of America; 3 Translational Research Institute for Metabolism and Diabetes, Orlando, Florida, United States of America; 4 University of Bath, Department of Health, Bath, United Kingdom; 5 Sanford-Burnham Medical Research Institute, Orlando, Florida, United States of America; Maastricht University Medical Center, The Netherlands

## Abstract

**Aims:**

To assess the determinants of exercise training-induced improvements in glucose control (HbA_1C_) including changes in serum total adiponectin and FFA concentrations, and skeletal muscle peroxisome proliferator-activated receptor-γ coactivator-1α (PGC-1α) protein content.

**Methods:**

A sub-cohort (n = 35; 48% men; 74% Caucasian) from the HART-D study undertaking muscle biopsies before and after 9 months of aerobic (AT), resistance (RT), or combination training (ATRT).

**Results:**

Changes in HbA_1C_ were associated with changes in adiponectin (r = −0.45, *P* = 0.007). Participants diagnosed with type 2 diabetes for a longer duration had the largest increase in PGC-1α (r = 0.44, *P* = 0.008). Statistical modeling examining changes in HbA_1C_ suggested that male sex (*P* = 0.05), non-Caucasian ethnicity (*P* = 0.02), duration of type 2 diabetes (r = 0.40; *P*<0.002) and changes in FFA (r = 0.36; *P*<0.004), adiponectin (r = −0.26; *P*<0.03), and PGC-1α (r = −0.28; *P* = 0.02) explain ∼65% of the variability in the changes in HbA_1C_.

**Conclusions:**

Decreases in HbA_1C_ after 9 months of exercise were associated with shorter duration of diabetes, lowering of serum FFA concentrations, increasing serum adiponectin concentrations and increasing skeletal muscle PGC-1α protein expression.

**Trial Registration:**

ClinicalTrials.gov NCT00458133

## Introduction

Type 2 diabetes is as much a disease of disordered lipid metabolism as a disease of abnormal glucose metabolism [1]. Failure to balance skeletal muscle lipid uptake (free fatty acids, FFA) and storage in intracellular triacylglycerol with oxidation in mitochondria is implicated in impaired insulin action [2]. Individuals with type 2 diabetes have a reduced plasma adiponectin concentrations [3], reduced number of mitochondria [4], and lower skeletal muscle gene/protein expressions of peroxisome proliferator-activated receptor-γ coactivator-1α (PGC-1α), a key regulator of mitochondrial biogenesis [5] and oxidative metabolism [6]. While increased PGC-1α has been observed with acute exercise in humans [7], studies examining the effects of chronic exercise training on adiponectin and PGC-1α and their relationship to the change in glycemic control (HbA_1C_) in individuals with type 2 diabetes are absent.

We recently demonstrated in the Health Benefits of Aerobic and Resistance Training in individuals with type 2 Diabetes (HART-D) study that 9 months of combined aerobic (AT) and resistance (RT) training significantly reduced HbA_1C_ levels in individuals with type 2 diabetes [8]. The HART-D study also included AT only and RT only training groups that, together with the combined AT and RT training group, provide a unique opportunity to examine the influence of factors known to influence glycemic control, specifically serum FFA and adiponectin, skeletal muscle PGC-1α protein content, and anthropometric/demographic measures. We therefore hypothesized that the change in HbA_1C_ after exercise would be independently associated with serum FFA and adiponectin and the activation of skeletal muscle mitochondrial biogenesis pathways as determined by PGC-1α protein content.

## Methods

**Figure 1:**
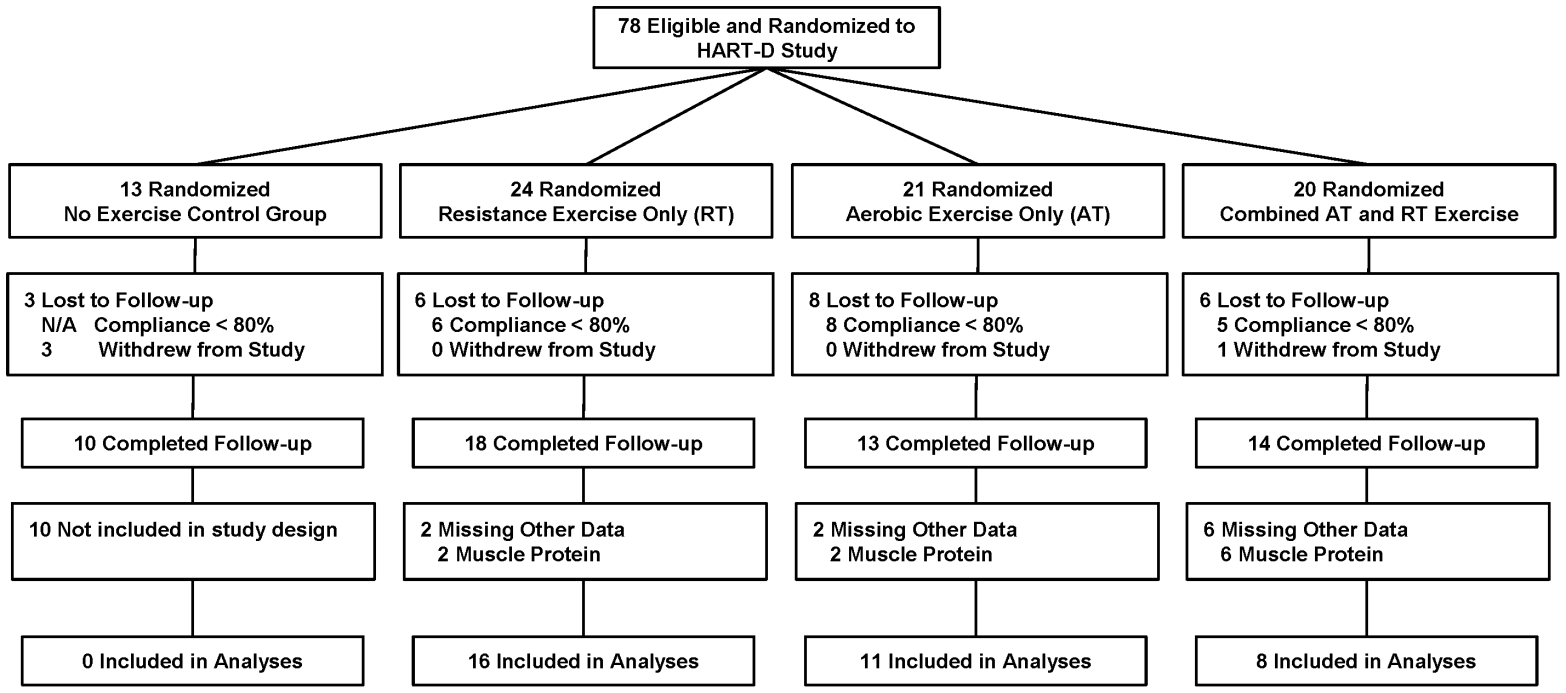
Consort diagram.

Seventy-eight participants from the previously reported HART-D study volunteered for and were enrolled into an ancillary study that included the collection of muscle samples at baseline and after 9 months of intervention (Figure 1). Detailed methods as well as inclusion/exclusion criteria are provided in the main outcomes paper [8]]. Briefly, 262 sedentary individuals with type 2 diabetes (HbA_1C_ 6.5% to 11%, inclusive) aged 30 to 75 years were enrolled into the study. Volunteers were excluded if body mass index was ≥48 kg/m^2^, blood pressure was ≥160/100 mmHg, fasting triglycerides were ≥500 mg/dL, urine protein was >100 mg/dL, or serum creatinine was >1.5 mg/dL. In addition, volunteers were excluded if they used an insulin pump or had a history of stroke, advanced neuropathy or retinopathy, or any other serious medical condition that prevented them from adhering to the protocol or exercising safety. Of the original 78 participants, 23 were lost to follow-up due to non-compliance (<80% attendance) with the study protocol leaving a total of 55 participants with both baseline and follow-up data. An additional 10 participants randomized to the control group were excluded from these analyses. An additional 10 participants' samples did not have protein extracted. The final group of 35 participants (17 males and 18 females) aged 57.0±7.7 y are included in the present analysis. The follow-up bloods draw and muscle biopsy for this ancillary project occurred approximately 1 week after their last exercise bout. The study was approved by the Pennington Biomedical Research Center institutional review board and written informed consent was obtained prior to study screening. The protocol for this trial and supporting CONSORT checklist are available as supporting information; see Checklist S1 and Protocol S1.

### Intervention

HART-D participants were randomized to 9 months of aerobic training (AT), resistance training (RT), a combination of both (ATRT), or a non-exercise control group. The AT group exercised 3–4 times per week at 50–80% of VO_2peak_ for a total energy expenditure of 12 kcal⋅kg^−1^⋅wk^−1^. The RT group completed 3 days per week of 2 sets of 4 arm exercises, 3 sets of 3 leg exercises, and 2 sets of back extension and abdominal crunch. The ATRT group completed 3 sessions of AT (10 kcal⋅kg^−1^⋅wk^−1^) and 2 sessions of RT (1 set each of the RT exercises) per week in conjunction with the Federal Physical Activity Guidelines [9]. Both exercise modalities were progressive in nature whereby the treadmill speed and grade was increased to maintain the appropriate intensity, reported in metabolic equivalent tasks (METS) [10] and the weight lifted on each RT exercise was increased when a participant could lift 12 repetitions on 2 consecutive sessions.

### VO_2max_ and body composition

Maximal cardiorespiratory fitness (VO_2max_) was assessed at baseline and after 9 months of intervention on a treadmill (Trackmaster 425, Newton, KS) with respiratory gases analyzed using a True Max 2400 Metabolic Cart (Parvomedics, Salt Lake City, UT). Peak oxygen uptake (VO_2peak_) was expressed relative to fat-free mass (mL⋅kg FFM^−1^⋅min^−1^). Body composition was measured by DXA (QDR 4500A, Hologic, Inc. Waltham, MA).

### Blood analyses

Blood was collected by venipuncture in the morning after a 10-hour fast at baseline and after the intervention. Blood samples were placed into pre-chilled red-top tubes (serum FFA and adiponectin) or EDTA collection tubes (plasma HbA_1C_) and transported to the laboratory on ice (FFA and HbA_1C_). The blood samples for FFA and HbA_1C_ were cold centrifuged and the media was immediately analyzed for FFA and HbA_1C_ (Beckman Coulter DXC600 Pro; Beckman Coulter Inc., Brea, CA). Blood collected for total serum adiponectin (Linco, St. Charles, MO) concentrations were transferred to the laboratory at room temperature, centrifuged, and serum was stored at −80°C until run in a single batch at the end of the trial. The intra-assay variation for HbA_1C_, FFA, and adiponectin was ±2.84%, ±0.75%, and ±6.21%, respectively.

### Muscle analysis

Muscle samples were obtained under local anaesthesia from the *vastus lateralis* using the Bergstrom technique and immediately snap frozen. Samples were homogenized by Kontes Duall tissue grinders in RIPA buffer with a cocktail of protease and phosphatase inhibitors (Sigma, St. Louis, MO). Total protein content was quantified by BCA assay (Pierce, Rockford, IL) and 25 uL was added to each gel for determination of PGC-1α protein content. PGC-1α protein content quantified relative to GAPDH (Abcam, Cambridge, MA) was determined by Western blot using an Odyssey 9120 Imaging System (LI-COR, Lincoln, NE) after probing with goat anti-mouse IgG Alexa Fluor 680 (Invitrogen, Carlsbad, CA). No data are available for the intra-assay variability in PGC-1α or GAPDH protein content as replicates were not run to preserve the remaining muscle tissue.

### Randomization and blinding

After signing the informed consent for the ancillary study, volunteers had baseline blood drawn and a muscle biopsy performed. Volunteers were later randomized to an intervention group according to the randomization schema within the main HART-D trial resulting in unequal group randomization. The nature of this intervention study prevents blinding of the exercise intervention personnel. However, every effort was made to maintain blinding of the assessment staff from the participant's intervention group.

### Statistical analysis

Analysis of variance (ANOVA) was used to assess baseline group differences (JMP 9.0.2, SAS Institute, Inc., Cary, NC) and dichotomous variables were examined using a χ^2^ test. All exercise groups were collapsed into a single group for the remaining analyses since no effect of exercise group was observed in the final model (*P* = 0.15), and the addition of exercise group to the final model did not influence the other factors in the model. Linear regression (Pearson r) was used to determine the relationships among change (post-pre) scores. Baseline-adjusted changes in anthropometric data, HbA_1C_, PGC-1α protein content, and fasting adiponectin and free-fatty acids were determined using analysis of covariance (ANCOVA) with group differences analyzed using Student-t post-hoc tests. In addition, ANCOVA was used to examine the relationships between changes in HbA_1C_ and changes in independent determinants (semi-partial correlation coefficients (r)). To determine the relationship between changes in HbA_1C_ and changes in FFA, adiponectin, and PGC1a content, we first adjusted for baseline HbA_1C_, sex, ethnicity, and duration of diabetes; all variables that were adjusted for in the main outcomes paper [8]. Furthermore, we aimed to determine whether weight, body composition and/or fitness (VO_2peak_) further explained the change in HbA_1C_ or removed any of the existing parameters from the model. Statistical significance was set at *P*<0.05 and data are reported as mean (95%CI) unless noted otherwise.

## Results

The average compliance for all exercise groups was 95.0±6.0% (mean±SD; range = 80.6% to 100.0%). From Month 2 to Month 9, estimated average METS in AT (AT = 5.7±1.0 to 7.2±1.0 METS and ATRT = 5.0±0.7 to 5.8±1.0 METS) and total weight lifted during RT (RT = 56,422±17,316 to 73,602±25,631 lbs and ATRT = 17881±6345 to 23,909±9,532 lbs) increased similarly to those reported in the main outcomes paper [8].

Baseline data for age, sex, diabetes duration, HbA_1C_ body composition, and VO_2peak_ (ml/kg FFM) in this subset of HART-D participants were similar to the main study for participants with an overall compliance ≥80% (all *P*>0.18). However, the percent of Caucasian participants was higher in this subset compared to the main study (74.3% vs. 56.7%, respectively, *P* = 0.045). Baseline data and baseline-adjusted treatment effects are presented in Table 1. Age was different across groups at baseline (*P* = 0.02). After training, change in VO_2peak_ was not significantly different between intervention groups in this cohort. Body weight was lower after AT compared with RT (*P*<0.05) due to a tendency for RT to increase FFM (*P* = 0.05). The baseline-adjusted change in HbA_1C_ in this cohort was independent of treatment group (*P* = 0.29). The change in HbA_1C_ adjusted for baseline HbA_1C_, age, ethnicity and type 2 diabetes duration, were similar to those in the larger cohort [8] albeit, not significant in this subset (exercise group effect, *P* = 0.60; −0.15% (−0.58%, 0.27%), −0.34% (−0.84%, 0.15%), and −0.49%(−1.03%, 0.05%) for RT, AT, and ATRT, respectively). PGC-1α response tended to differ by intervention group (*P* = 0.08; Table 1).

**Table 1:** Baseline participant characteristics and baseline adjusted changes in anthropometric data, HbA_1C_, PGC-1α protein content, and fasting adiponectin and free-fatty acids after 9 months of exercise.

	RT		AT		ATRT		*P*-value	
	Baseline	Change	Baseline	Change	Baseline	Change	Baseline	Change
n	16		11		8			
Age, y	60.9 (7.6)		53.8 (6.6)		53.6 (6.4)		0.02^a^	
Sex, male %	8 (50.0)		6 (54.6)		3 (37.5)		0.75	
Ethnicity, Caucasian %	12 (75.0)		9 (81.8)		5 (62.5)		0.64	
Type 2 Diabetes Duration, y	9.5 (6.9)		6.0 (4.8)		5.9 (3.4)		0.20	
Body Weight, kg	98.5 (15.1)	0.5 (−0.7, 1.6)	90.5 (11.0)	−1.6 (−3.0, −0.2)	100.5 (22.3)	−1.3 (−2.9, 0.3)	0.32	<0.05^a^
Fat Mass, %	37.4 (8.5)	−0.9 (−1.8, 0.0)	31.7 (8.8)	−0.3 (−1.4, 0.8)	38.4 (6.6)	−0.9 (−2.1, 0.4)	0.14	0.67
FFM, kg	61.6 (11.5)	1.3 (0.4, 2.2)	61.6 (9.3)	−0.3 (−1.4, 0.8)	62.3 (15.6)	−0.1 (−1.4, 1.2)	0.99	0.05
VO_2peak_, mL⋅kg FFM^−1^⋅min^−1^	31.3 (4.6)	−0.1 (−2.1, 1.9)	34.7 (6.6)	2.0 (−0.5, 4.5)	30.3 (3.3)	0.6 (−2.2, 3.5)	0.13	0.43
HbA_1C,_ %	7.1 (1.1)	0.0 (−0.3, 0.4)	7.4 (1.4)	−0.2 (−0.7, 0.2)	6.9 (0.7)	−0.4 (−0.9, 0.1)	0.61	0.29
FFA, mmol/L	0.56 (0.21)	−0.00 (−0.11, 0.11)	0.66 (0.22)	0.04 (−0.09, 0.17)	0.54 (0.24)	−0.07 (−0.22, 0.08)	0.47	0.56
Adiponectin, µg/mL	8.1 (4.2)	−0.9 (−2.9, 1.0)	6.7 (2.9)	0.3 (−2.0, 2.6)	6.4 (3.9)	−1.8 (−4.5, 0.9)	0.47	0.47
PGC-1α/GAPDH, au	0.04 (0.04)	0.02 (0.00, 0.03)	0.05 (0.04)	0.00 (−0.01, 0.02)	0.06 (0.04)	−0.01 (−0.03, 0.01)	0.53	0.08

All baseline data are means (SD), n (%), and baseline-adjusted change scores are mean (95%CI). Abbreviations: RT, resistance training; AT, aerobic training; ATRT, combination of aerobic and resistance training; VO_2_ volume of oxygen consumed in mL per kg fat-free mass (FFM) per minute; HbA_1C,_ glycated hemoglobin A_1c_; PGC-1α/GAPDH, peroxisome proliferator-activated receptor-γ coactivator-1α adjusted for glyceraldehyde 3-phosphate dehydrogenase protein content; FFA, free fatty acid.

a RT group significantly greater than greater than AT. Blank cells are not applicable.

Change in fasting serum adiponectin was inversely associated with the change in HbA_1C_ (r = −0.45; *P* = 0.007). Change in HbA_1C_ was not related to change in fasting serum FFA (r = 0.25; *P* = 0.15) or change in PGC-1α protein content (r = 0.05; *P* = 0.80). Participants with longer durations of type 2 diabetes had the largest increase in PGC-1α (r = 0.44; *P* = 0.008). Last, change in VO_2peak_ was inversely related with age (r = −0.36; *P*<0.04). No other significant relationships exist.

In the process of building our statistical model, we examined the effect of change in serum FFA and adiponectin, and PGC-1α protein content, separately, on changes in HbA_1C_ after adjusting for baseline HbA_1C_, sex, and ethnic group. The changes in serum FFA (r = 0.33; *P*<0.02) and adiponectin (r = −0.30; *P*<0.03) were associated with the changes in HbA_1C_; however, change in PGC-1α protein content did not reach statistical significance (r = 0.17; *P* = 0.24).

**Figure 2:**
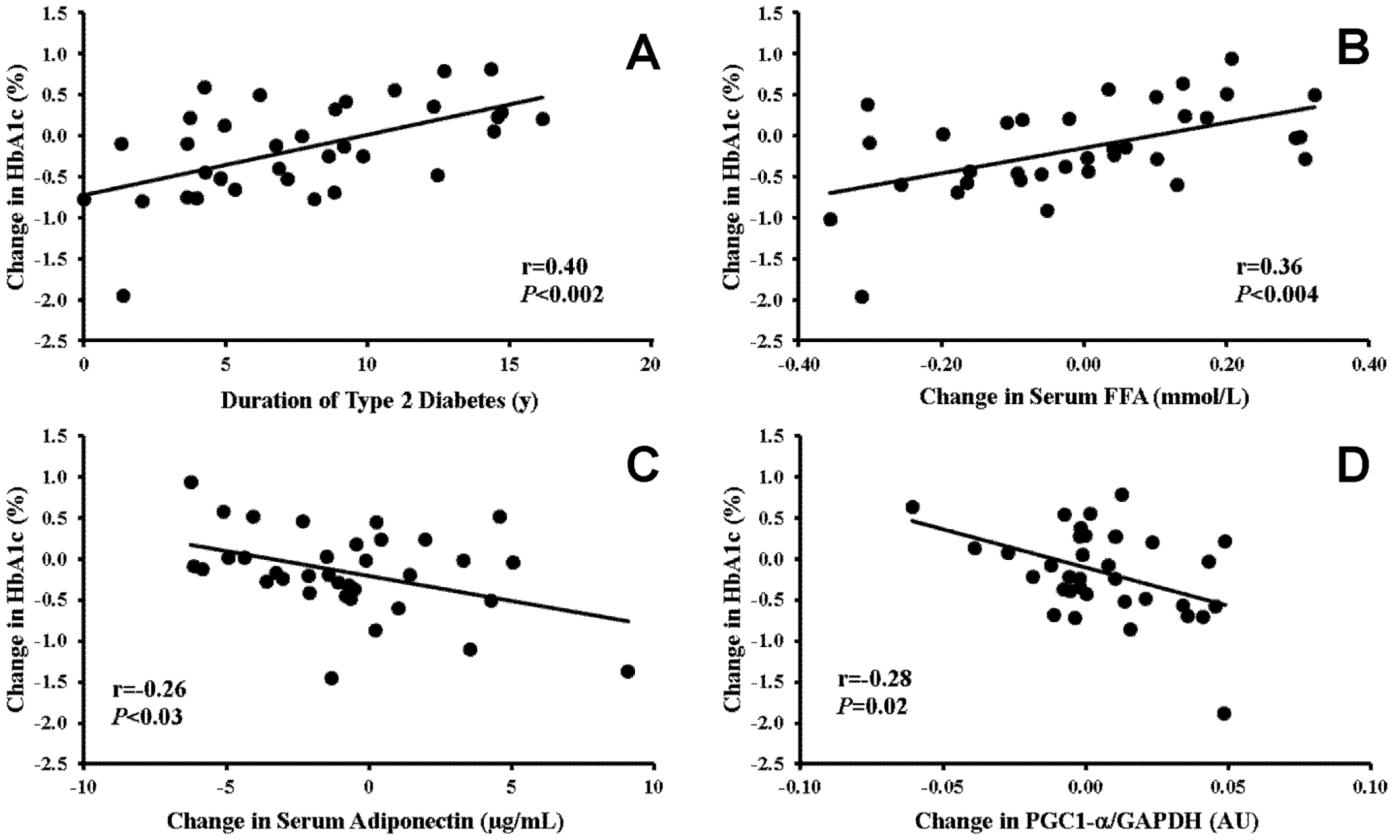
Independent effects (semipartial correlation coefficients (r)) of type 2 diabetes duration (A) and change in serum FFA (B), serum adiponectin (C), and intramuscular peroxisome proliferator-activated receptor-γ coactivator-1α (PGC-1α) protein content adjusted for glyceraldehyde 3-phosphate (GAPDH) protein content (D) on change in HbA_1C_ (%) after 9 months of exercise after adjusting for baseline HbA_1C_, sex, and ethnicity. Change in HbA_1C_ is reported as residuals (A–D) from the regression model (Δ HbA_1C_ = −.48(baseline HbA_1C_)−0.23(sex)+0.32(ethnic group)+0.07(type 2 diabetes duration)+1.54(ΔFFA)−0.06(Δadiponectin)−9.29(ΔPGC-1α)+2.52). Units for the prediction equation are similar to those in each panel; sex, 1 = men and 0 = women; ethnic group, 1 = Caucasian and 0 = Non-Caucasian.

After adjusting for baseline HbA_1C,_ changes in HbA_1C_ were related to type 2 diabetes duration (r = 0.40, *P*<0.002; Figure 2A), changes in fasting serum FFA (r = 0.36, *P*<0.004; Figure 2B) and, adiponectin (r = −0.26, *P*<0.03; Figure 2C) levels, and skeletal muscle PGC-1α protein (r = −0.28, *P* = 0.02; Figure 2D) independent of sex (−0.09% (−0.36%, 0.17%) vs. −0.55% (−0.92%, −0.17%); women vs. men, respectively; *P* = 0.05) and ethnicity (−0.00% (−0.23%, 0.23%) vs. −0.64% (−1.09%, −0.19%), Caucasian vs. non-Caucasian, respectively; *P* = 0.02). Together, these factors explained ∼65% of the variance in the change in HbA_1C_. The regression equation for the model is presented below. Changes in fitness (VO_2peak_) and body composition did not influence this model.
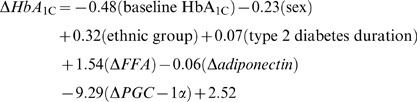



## Conclusions

The novel finding of the present investigation was that the changes in HbA_1C_ after 9 months of exercise were independently associated with the duration of type 2 diabetes and changes in serum FFA and negatively associated with changes in serum adiponectin and skeletal muscle PGC-1α.

The change in free-fatty acid concentration was a major determinant of the change in glycemic control. Free-fatty acid accumulation in the circulation may be the result of an imbalance between skeletal muscle uptake and oxidation and defects in adipose tissue insulin signaling [2]. Lipid oversupply to skeletal muscle may cause insulin resistance through accumulation of incompletely oxidized lipid species [11]. In the present investigation, improved balance between muscle lipid storage and oxidation (less lipid species storage), as suggested by a decrease in serum FFA, may be an important contributor to the improvement in HbA_1C_.

We observed a strong inverse relationship between the changes in adiponectin and the changes in HbA_1C_. Civitarese et al. recently uncovered a pathway by which adiponectin increases skeletal muscle PGC-1α, mitochondrial number and oxidative capacity [12]. Adiponectin may also have beneficial effects, independent of PGC-1α, by enhancing ceramidase activity, thus reducing the amount of insulin desensitizing ceramides [13]. Our results demonstrate a clinically relevant situation in which the change in serum adiponectin was associated with an improvement in HbA_1C_.

A recent review called for more effective and sophisticated exercise prescriptions for the improvement of glucose control through increasing intramuscular PGC-1α [14]. Studies demonstrate increased PGC-1α with aerobic training [7], while others show no effect or improvements with resistance training [15], [16]. We tested different exercise modalities and only resistance training had a tendency to increase PGC-1α content. Additionally, the change in PGC-1α was greater in those with longer duration of type 2 diabetes. In our final model, a greater increase in PGC-1α and a shorter duration of type 2 diabetes were independently associated with improved glycemic control. The main results from the HART-D trial together with this ancillary study suggest that: 1) a program of combined aerobic and resistance training has the greatest effect on HbA_1C_; 2) resistance training may potentiate a greater change in PGC-1α, and therefore, HbA_1C;_ and 3) individuals who start an exercise program soon after diagnosis may see a larger effect on HbA1C levels.

Our investigation is limited by a small sample size with almost 50% of the participants assigned to the resistance training group. However, we had adequate power to detect independent effects of substrate (FFA), hormone (adiponectin) and muscle (PGC-1α) changes on glycemic control after collapsing the exercise groups. Drug changes were not controlled during the intervention period resulting in a limitation whereby a tendency to reduce diabetes medications after the combination intervention reduces the effect on change in HbA_1C_ levels. Furthermore, dietary assessments were taken at baseline and follow-up by food frequency questionnaire thus limiting our ability to determine the impact of dietary modifications such as changes in caloric intake and dietary composition. Finally, research suggests that GAPDH gene transcription may be sensitive to treatments that may influence PGC-1α protein content, including exercise-induced changes in insulin sensitivity [17]. However, analysis of the crude and GAPDH adjusted PGC-1α data yielded similar results providing evidence that the effects noted in this manuscript were the result of the exercise intervention on PGC-1α specifically and not secondary changes in GAPDH. The primary strengths are a well-controlled exercise training study with high adherence rates and a wide range of changes in the dependent and independent variables.

In summary, exercise in individuals with type 2 diabetes should be initiated soon after diagnosis and include training programs aimed at improving plasma substrate availability, endocrine function, and skeletal muscle factors shown to improve glycemic outcomes.

## Supporting Information

Checklist S1 **CONSORT Checklist.**
(DOC)

Protocol S1 **Trial Protocol.**
(PDF)

## References

[1] McGarry JD (1992) What if Minkowski had been ageusic? An alternative angle on diabetes. Science 258: 766–770. 1439783 10.1126/science.1439783

[2] Ravussin E, Smith SR (2002) Increased fat intake, impaired fat oxidation, and failure of fat cell proliferation result in ectopic fat storage, insulin resistance, and type 2 diabetes mellitus. Ann N Y Acad Sci 967: 363–378. 12079864 10.1111/j.1749-6632.2002.tb04292.x

[3] Hotta K, Funahashi T, Arita Y, Takahashi M, Matsuda M, et al (2000) Plasma concentrations of a novel, adipose-specific protein, adiponectin, in type 2 diabetic patients. Arterioscler Thromb Vasc Biol 20: 1595–1599. 10845877 10.1161/01.atv.20.6.1595

[4] Ritov VB, Menshikova EV, He J, Ferrell RE, Goodpaster BH, et al (2005) Deficiency of subsarcolemmal mitochondria in obesity and type 2 diabetes. Diabetes 54: 8–14. 15616005 10.2337/diabetes.54.1.8

[5] Mootha VK, Lindgren CM, Eriksson KF, Subramanian A, Sihag S, et al (2003) PGC-1alpha-responsive genes involved in oxidative phosphorylation are coordinately downregulated in human diabetes. Nat Genet 34: 267–273. 12808457 10.1038/ng1180

[6] Puigserver P, Spiegelman BM (2003) Peroxisome proliferator-activated receptor-gamma coactivator 1 alpha (PGC-1 alpha): transcriptional coactivator and metabolic regulator. Endocr Rev 24: 78–90. 12588810 10.1210/er.2002-0012

[7] Pilegaard H, Saltin B, Neufer PD (2003) Exercise induces transient transcriptional activation of the PGC-1alpha gene in human skeletal muscle. J Physiol 546: 851–858. 12563009 10.1113/jphysiol.2002.034850 PMC2342594

[8] Church TS, Blair SN, Cocreham S, Johannsen N, Johnson W, et al (2010) Effects of aerobic and resistance training on hemoglobin A1c levels in patients with type 2 diabetes: a randomized controlled trial. JAMA 304: 2253–2262. 21098771 10.1001/jama.2010.1710 PMC3174102

[9] Services USDoHaH (2008) 2008 Physical Activity Guidelines for Americans. Rockville, MD: U.S. Department of Health and Human Services, Office of Disease Prevention and Health Promotion.

[10] (2006) American College of Sports Medicine. ACSM's Guidelines for Exercise Testing and Prescription. Philadelphia, PA: Lippincott Williams & Williams.

[11] Koves TR, Ussher JR, Noland RC, Slentz D, Mosedale M, et al (2008) Mitochondrial overload and incomplete fatty acid oxidation contribute to skeletal muscle insulin resistance. Cell Metab 7: 45–56. 18177724 10.1016/j.cmet.2007.10.013

[12] Civitarese AE, Ukropcova B, Carling S, Hulver M, DeFronzo RA, et al (2006) Role of adiponectin in human skeletal muscle bioenergetics. Cell Metab 4: 75–87. 16814734 10.1016/j.cmet.2006.05.002 PMC2671025

[13] Holland WL, Miller RA, Wang ZV, Sun K, Barth BM, et al (2011) Receptor-mediated activation of ceramidase activity initiates the pleiotropic actions of adiponectin. Nat Med 17: 55–63. 21186369 10.1038/nm.2277 PMC3134999

[14] Benton CR, Wright DC, Bonen A (2008) PGC-1alpha-mediated regulation of gene expression and metabolism: implications for nutrition and exercise prescriptions. Appl Physiol Nutr Metab 33: 843–862. 18923559 10.1139/H08-074

[15] Layne AS, Nasrallah S, South MA, Howell ME, McCurry MP, et al (2011) Impaired muscle AMPK activation in the metabolic syndrome may attenuate improved insulin action after exercise training. J Clin Endocrinol Metab 96: 1815–1826. 21508135 10.1210/jc.2010-2532 PMC3100747

[16] Heilbronn LK, Gan SK, Turner N, Campbell LV, Chisholm DJ (2007) Markers of mitochondrial biogenesis and metabolism are lower in overweight and obese insulin-resistant subjects. J Clin Endocrinol Metab 92: 1467–1473. 17244782 10.1210/jc.2006-2210

[17] Alexander-Bridges M, Dugast I, Ercolani L, Kong XF, Giere L, et al (1992) Multiple insulin-responsive elements regulate transcription of the GAPDH gene. Adv Enzyme Regul 32: 149–159. 1386708 10.1016/0065-2571(92)90014-q

