# Supplementary material for: Determinants of the Changes in Glycemic Control with Exercise Training in Type 2 Diabetes: A Randomized Trial
Source: PLoS One. 2013 Jun 21;8(6):e62973. doi: 10.1371/journal.pone.0062973 (PMC3689775; doi:10.1371/journal.pone.0062973)
Supplement: Protocol S1 — Trial Protocol. (PDF) [file pone.0062973.s002.pdf]

Mitochondrial Adaptations to Aerobic and Resistance Training in Diabetes II (MARTi II)

Lauren M. Sparks, PhD, Principal Investigator
Timothy S. Church, MD, MPH, PhD, Co-investigator
Neil Johannsen, PhD, Co-investigator
Cedric Moro, PhD, Co-investigator
Sudip Bajpeyi, PhD, Co-investigator
Kevin Conley, PhD, Co-investigator

PBRC Institutional Review Board
FWA 00006218
Approved On 1-30-08
Signature

A. Specific Aims and Hypothesis

Diabetes is as much a disease of disordered lipid metabolism as a disease of glucose metabolism (1). Defects in lipid metabolism are present early in the course of the disease and disturbances in lipid metabolism are amplified as glucose control deteriorates. Disordered lipid metabolism is important for the pathophysiology of insulin resistance and type 2 diabetes (T2DM). Both VLDL-triglyceride and NEFA are elevated in T2DM. The uptake of lipids in skeletal muscle must be balanced by storage in intracellular TAG pools or oxidation in mitochondria. An imbalance between net uptake and oxidation leads to the accumulation of 'toxic' lipid intermediates that impair insulin action and glucose metabolism. This state has been referred to as 'lipotoxicity' or 'ectopic' fat (2). It is well documented that individuals with T2DM have both a reduced number of mitochondria and dysfunctional mitochondria (3, 4). Given that mitochondria are the primary site of lipid oxidation and that defects in mitochondrial function and fat oxidation are early events in development of T2DM (5, 6), our basic premise is that exercise training will increase mitochondrial function and lipid oxidation inT2DM.

Both aerobic and resistance training can be hypothesized to improve mitochondrial function / fatty acid oxidation; however, this hypothesis remains largely untested, particularly in individuals with T2DM. Furthermore, by increasing both muscle mass and mitochondrial function, combined aerobic and resistance training may have more benefit than either intervention alone.

This proposed mechanistic study of the training effects on mitochondrial function and lipid oxidation in T2DM is a prospective ancillary study to the NIDDK-funded Health Benefits of Aerobic and Resistance Training in individuals with type 2 diabetes (HART-D). HART-D was reviewed by the PRDP review group in February 2006 (priority score = 141, percentile 4.9), and was funded in July 2006. In HART-D, sedentary women and men with T2DM will be randomly assigned to an aerobic exercise training only group (AT), a resistance training only group (RT), a combination of aerobic plus resistance training (AT+RT) or a stretching and relaxation group (SR) to serve as a control. The primary outcome of the parent trial is hemoglobin A1c (HbA1c). The AT individuals will participate in 3 or 4 training sessions each week for 9 months progressing to a total energy expenditure of 12 kcal/kg/week (KKW), which is an exercise dose consistent with the current public health recommendations for physical activity for individuals with T2DM. (7, 8) The target exercise intensity will be 50%-80% of baseline VO_2 max. The RT group will participate in 3 sessions per week (30-45 minutes each), which focuses on large muscle groups at an intensity of 40-80% of 1-repetition maximum for the given exercise. Individuals in the AT+RT group will receive both a reduced aerobic training regimen of progression to a total energy expenditure of 10KKW and a reduced resistance-training regimen of 2 sessions per week at 20 minutes per session. This combination regimen represents the exact exercise recommendations of the American College of Sports Medicine (ACSM) and the American Diabetes Association (ADA) (7, 8). Simply stated, HART-D will examine if a hybrid program of AT+RT is superior to AT alone (or RT alone) in reducing HbA1c and if a RT has comparable benefits in lowering HbA1c to a standard AT program. At the time of this submission, HART-D is in the first year of its four years of trial activities with 64 participants currently randomized.

We propose to measure mitochondrial function in a subset of HART-D participants (n= 20 aerobic trained, 20 resistance trained, 20 combined resistance / aerobic trained and 20 stretching and relaxation controls) to test **the hypothesis that exercise training will increase mitochondrial function and lipid oxidation in T2DM.**

Specific Aim – to measure mitochondrial function and fat oxidation *(ex vivo studies)* in a subset of participants enrolled in HART-D to test the hypotheses that:

- Mitochondrial function and fat oxidation will increase with both aerobic and resistance training relative to the control group and are <u>additive</u> when combined.
- Baseline mitochondrial function and fat oxidation are inversely associated with baseline plasma NEFAs.

This will be the first randomized controlled study comparing the effectiveness of aerobic vs. resistance training to reverse defects in mitochondrial biogenesis and lipid oxidation in T2DM under rigorously controlled conditions in the clinic and with state-of-the-art non-invasive ex vivo measures of mitochondrial function.

We will determine the mechanism(s) by which exercise training improves lipid oxidation in T2DM by measuring key components of the lipid oxidation system in skeletal muscle *(ex vivo studies)*. Our hypothesis is that each of the following systems 'improves' with exercise training and increases coupling of lipid uptake to *complete* mitochondrial oxidation:

1. serum adiponectin	[RIA]	*Sparks*
2. adiponectin receptors 1/2	[mRNA]	*Sparks*
3. PGC1α	[Western immunoblotting]	*Sparks / Gettys*
4. Mitochondrial mass	[mtDNA, citrate synthase and EM analysis]	*Sparks / Sellers*
5. ETC activity	[isolated mitochondria from skeletal muscle]	*Sparks*
6. Mitochondrial lipid oxidation	[homogenate of skeletal muscle]	*Sparks*
7. Topology of lipid droplets **[E]**	[association of lipid droplets with mitochondria - analysis of EM]	
	[lipid coat proteins by Western blotting]	*Moro / Burke*
8. mitochondrial lipid transport **[E]**	[Western immunoblotting $\pm$ confocal IHC]	*Sparks / Burke*
	**[E] = exploratory**	

The purpose of this aim is not to identify the toxic lipid species that might amplify defects in insulin signaling and glucose metabolism, but to focus on the mechanisms by which resistance and aerobic training alter the structure of the lipid oxidation pathway in skeletal muscle of exercising type 2 diabetics. As an ancillary to a funded intervention trial HART-D, this study represents a unique, timely and cost effective opportunity to quickly test important hypotheses in a randomized, controlled, clinical trial setting using state-of-the-art measures and clinically relevant exercise interventions.

B. Background and Significance

B1. Defects in fat oxidation in T2DM Studies almost a decade ago (9, 10) showed that defects in fat oxidation and mitochondrial content were present in insulin resistance. As summarized by Kelley and Mandarino (11), metabolic flexibility is impaired in T2DM and obesity. The two key features of metabolic ***in***flexibility are decreased fat oxidation in the fasting state (i.e. higher RQ) and decreased insulin-stimulated glucose oxidation. Metabolic ***in***flexibility was originally defined as an inability to switch between the utilization of lipids during fasting to the utilization of glucose after a meal. We have recently found these same defects (I.e. metabolic inflexibility) in young men with a FH of T2DM (6) before the onset of hyperglycemia and independent of body fatness or visceral fat mass. At the whole body level, fat oxidation in response to a high fat diet is impaired in insulin resistant young men (12). We also found that increased physical activity accelerates the adaptation to a high fat diet (13), and this result has recently been replicated (14). Inactivity also reduces fat oxidation (15) and reduces mitochondrial content in skeletal muscle.

One surprising result of our work is that high fat diets decreased the transcription of genes required for mitochondrial biogenesis and fat oxidation (16). This result was replicated by Richardson et al. (17) who found that lipid/heparin infusions decreased PGC1a mRNA within hours. Conversely, suppression of FFA improves insulin action and increases PGC1a (18, 19). This suggests that

impairments in lipid oversupply changes the structure of muscle, specifically mitochondrial biogenesis and fat oxidation, in a way that might further impair lipid oxidation.

These defects in lipid oxidation are important for several reasons. First, reduced fat oxidation is hypothesized to result in increased fat gain in individuals consuming a high fat diet. Second, the accumulation of lipid intermediates [e.g. long chain coAs (20), DAGs (21), ceramides (22)] are increased and activate a variety of signaling systems such as PKC (23) leading to the inhibition of insulin signaling (24). This application focuses on the overall fat oxidation pathway, rather than specific lipid intermediates, because we hold the views that a) there is good evidence for defects in fat oxidation at multiple steps and b) that defects in the lipid oxidation are likely to result in the generation of multiple 'toxic' lipid intermediates. As such, interventions such as aerobic and resistance training are likely to abrogate many of these defects and reduce 'lipotoxicity' generally. **This application will focus on the known or hypothesized structural defects in mitochondria / fat oxidation with a general goal to compare / contrast the effects of aerobic and resistance training. As discussed in the methods, additional lab studies may be performed to measure specific lipid intermediates based on the results of the planned experiments or changes in the field of study as a whole.**

B2. Localizing the defect in fat oxidation There are several potential causes of the defects in fat oxidation in T2DM. Lipid supply to skeletal muscle is clearly excessive (increased FFA and TAG) and the increase in lipid supply is evidenced by increased intramyocellular lipid (25). This also suggests that lipid uptake is not impaired, and this is consistent with limb balance studies where the fractional extraction of FFA is high (~40%) and does not differ across insulin resistant / insulin sensitive states [David Kelley, personal communication]. Thus the defects in lipid oxidation are due to either continuous activation of glucose metabolism or structural / functional defects in the flux of lipid through oxidative pathways. There is some evidence that glycolytic pathways might be increased (9) but most of the attention in the last decade has been focused on potential defects in the capacity for fat oxidation. These are illustrated in **Figures B1 and B2**. Techniques marked with an asterisk * are proposed in this application and detailed in the methods.

Multiple defects along the fat oxidation pathway have been described in obesity, insulin resistance and T2DM, and a few of these will be discussed in more detail. First, numerous studies demonstrate that the product of ACC, malonyl CoA, is increased in the skeletal muscle in T2DM; this inhibits the activation of CPT I, considered by most to be the rate limiting step in fatty acid entry into mitochondria (26). When normalized to the oxidation of pyruvate, fatty acid oxidation is impaired in isolated mitochondria and this is associated with reduced fatty acid oxidation during low intensity exercise (27). Similarly, several studies have demonstrated that the oxidation of labeled fatty acids is reduced in skeletal muscle homogenates from obese individuals * (28, 29). This suggests that the fat oxidative machinery is reduced in content or is dysfunctional. This is consistent with data from Pima Indians where βHAD activity was tightly correlated to 24h fat oxidation (30), again suggesting that the capacity for fat oxidation determines whole body fat oxidation. Structural changes in the mitochondria [for example reduced lipid transport into the mitochondrial by CPT-I * or CD36 * (31)] are also possible *. Along these same lines, Tarnopolsky et al. recently described a reduced number of connections between TAG droplets and mitochondria in sedentary individuals; this improved with aerobic training * (32). TAG droplets are coated with the protein ADRP in skeletal muscle and increased with weight loss. The skeletal muscle protein content of ADRP * is reduced in insulin resistance/obesity, which along with other TAG / lipid coat proteins, might account for the change in their association with mitochondria (33). Importantly, Tarnopolsky et al. found a high correlation between the proportion of lipid droplets associated with mitochondria and fasting non-esterified (free) fatty acids (NEFAs). Along these same lines, they showed that the size of lipid droplets was increased in T2DM, and a combined exercise / weight loss intervention decreased the size of lipid droplet (34). The reduction in the size of the lipid droplet was one of the best correlates of the improvements in insulin sensitivity in that study. Skeletal muscle lipolysis is another potential site of dysregulation leading to lipotoxicity. However, there is currently little data on the regulation of TAG lipolysis in T2DM; this is an active area of research in our laboratory but will not be the focus of this investigation. Taken together, this data suggests that defects in fat oxidation occur at multiple steps along the lipid oxidation pathway; increased malonyl CoA, changes in the proteins coating

lipid droplets, reduced proximity of TAG droplets to mitochondria and changes in the concentrations of mitochondrial transport proteins, to name a few of our candidates. As we review in the next section, there is solid evidence that at least part of the defect in fat oxidation lies in a reduced mitochondrial number and function.

B3. Defects in mitochondrial mass and function in T2DM As noted above, early studies by Simoneau and Kelley described reduced oxidative capacities of skeletal muscle in T2DM (35). They hypothesized that an imbalance in the glycolytic to oxidative capacity is important in the pathogenesis of T2DM. Strong support for this hypothesis came from the functional studies of Petersen et al. who demonstrated that mitochondrial function was reduced in elderly subjects with insulin resistance (36) and in non-diabetic individuals with a family history of T2DM (5). This is consistent with the observed ultrastructural defects in the mitochondria of T2DM patients (37). Kelley also observed a reduction in the number of mitochondria, particularly in the sub-sarcolemmal region. Aging is associated with insulin resistance (36). As such, studies of mitochondrial function in aging may be relevant to the mitochondrial dysfunction seen in T2DM. Recent studies from Conley et al. demonstrate that healthy volunteers aged 60-70 have substantial defects in mitochondrial function as evidenced by a decrease in maximal ATP synthetic capacity (38) in a population similar to Petersen, et al. [see preliminary data **Figure C2**]. Taken together, this suggests that defects in mitochondria might underlie insulin resistance and impaired fat oxidation in T2DM.

B4. Localizing the defect in mitochondrial biogenesis in T2DM Important clues as to the signaling pathways involved in mitochondrial biogenesis came from the cloning of two critical genes: (PGC1a) and (NRF1). Together, these two nuclear receptor co-activators coordinately regulate a wide variety of

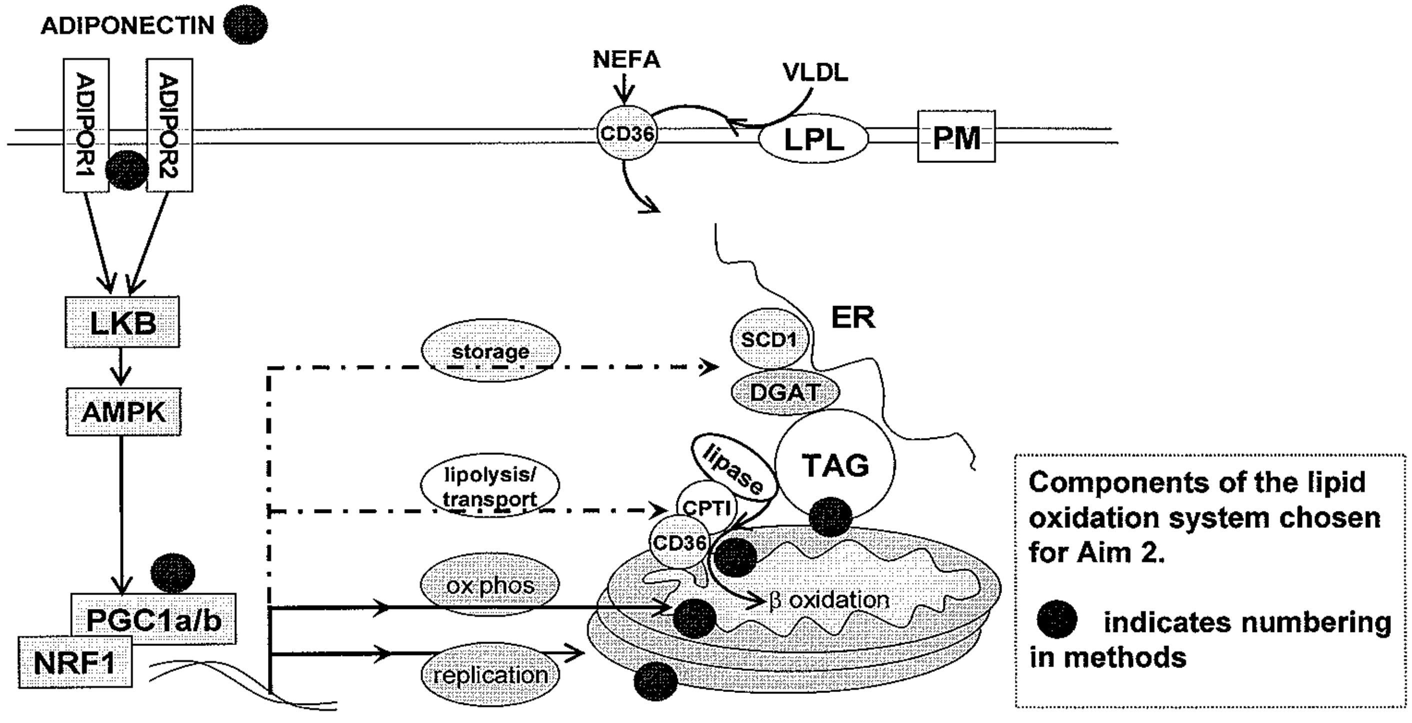

Figure B1 general schematic for lipid metabolism in mitochondria

skeletal muscle genes necessary for mitochondrial biogenesis (39), fiber type switching (40), reactive oxygen species scavenging {St-Pierre, 2006, Civitarese, 2006} and fatty acid oxidation (41, 42).

PGC1a lies upstream of and co-activates the nuclear receptor NRF1. Transgenic overexpression of PGC1a increases oxidative capacity in mice and also increases fat oxidation (43). Muscle contraction increases the protein content and activates PGC1a transcriptional events (44, 45). Importantly, contraction of insulin resistant muscles increases glucose transport, increases mitochondrial biogenesis and increases fatty acid oxidation (46) without improvements in upstream insulin signaling; whereas,

downstream insulin signaling was improved. This dissociation between contraction mediated improvements in lipid oxidation and continued upstream defects in insulin action supports our overarching hypothesis, namely that exercise improves mitochondrial function and increases lipid oxidation. Contraction also increases calcium signaling through calcineurin (47) to activate PGC1a, mitochondrial biogenesis and fat oxidation. cAMP signaling through PKA (40) is also important in PGC1a activation.

The primacy of PGC1a * and PGC1b to the defects in mitochondrial mass and function in T2DM is becoming more apparent (48, 49). Muoio and colleagues found that when muscle cells were transfected with PGC1a they were capable of not only increased fat oxidation (41), but importantly there was tighter 'coupling' of beta oxidation to TCA cycle oxidation *. This led to a reduction in intermediates of lipid metabolism and the hypothesis that these intermediates might contribute to the insulin resistant state. They proposed that mismatched β-oxidation and complete oxidation lead to insulin resistance * . Our hypothesis is that β-oxidation and complete oxidation will be increased by aerobic, resistance and especially the combined training, in T2DM.

The cellular sensor of energy status AMP-activated protein kinase (AMPK) activates PGC1a and mitochondrial biogenesis. Upstream of AMPK are two plasma membrane receptors, AdipoR1 * and R2 *. These novel receptors are activated by the adipose tissue secreted endocrine hormone adiponectin. Adiponectin is reduced in T2DM (50). Furthermore, the skeletal muscle adiponectin receptors are downregulated in the offspring of T2DM (51). Combined with recent data demonstrating that adiponectin knockout mice have reduced mitochondrial content and treatment of muscle cells *ex vivo* activates mitochondrial biogenesis and fat oxidation (42) adiponectin * and its receptors * serve as **Figure B2**

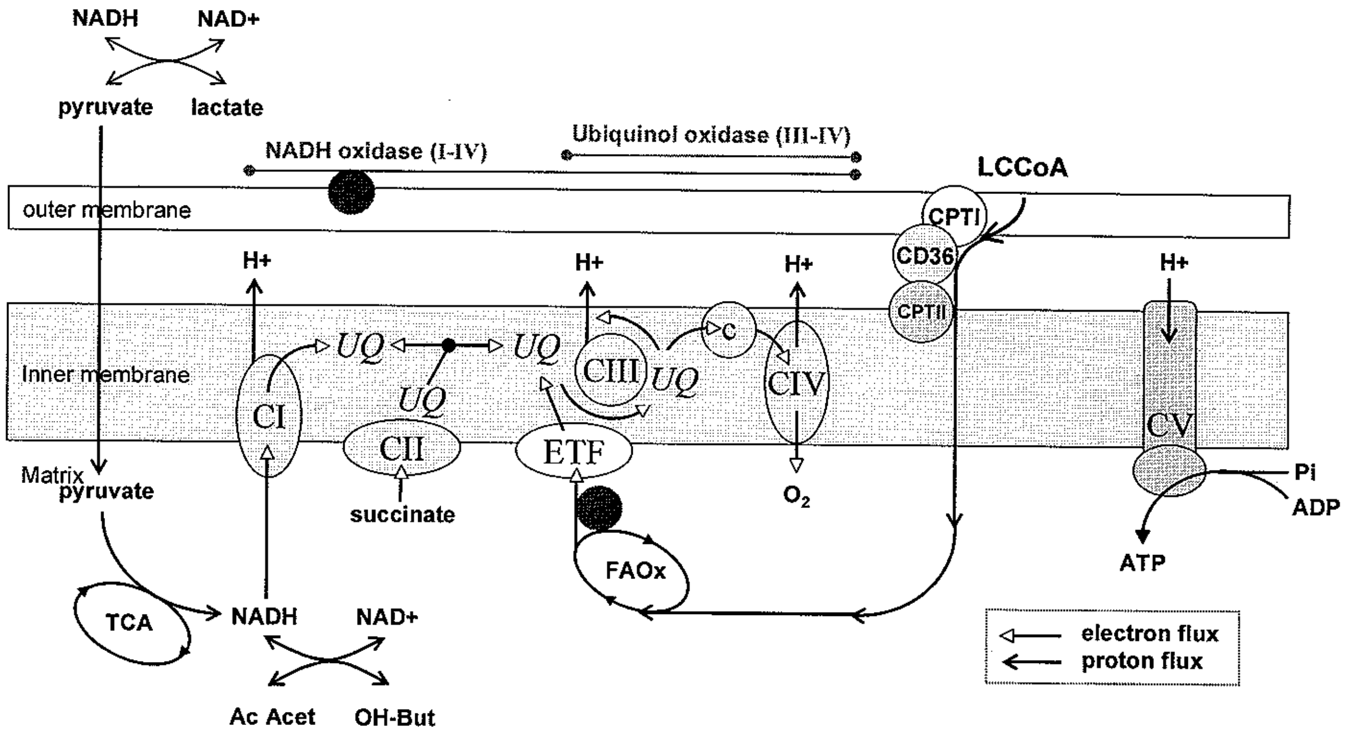

general schematic for lipid and glucose metabolism in mitochondria

solid candidates for the reduced mitochondrial mass and function seen in T2DM. Although we do not propose to measure AMPK protein / activity, using a protocol generously provided by Laurie Goodyear at the Joslin, we will save freeze-clamped tissue and homogenize that tissue in a phosphatase/protease inhibitor cocktail in on a monthly basis to reserve the possibility of measuring AMPK.

B5. Aerobic exercise training increases PGC1a and increases mitochondrial mass and the capacity for mitochondrial fat oxidation

As noted, above, aerobic exercise training increases muscle content and transcriptional co-activation of PGC1a. Mitochondrial mass increases with aerobic exercise training of sedentary non-diabetic individuals and numerous studies show an increase in muscle citrate synthase with aerobic training (32, 52). Concurrently, aerobic exercise training increases muscle fatty acid oxidation capacity (53). Kelley et al. showed minor defects in mitochondrial mass as measured by mtDNA and citrate synthase, but major defects in mitochondrial function as measured by ETC. Therefore, it is important to note differences in the methods used to assess mitochondrial function. For example, using sensitive measures of mitochondrial electron transport [ETC] chain activity, Kelley et al. showed that aerobic training combined with weight loss produces dramatic (+100%) changes in ETC activity in T2DM, but only modest changes in citrate synthase activity (+20%) and mtDNA content (+20%) (54). *This suggests that measures of mitochondrial function rather than mass, specifically ETC and an in vivo measure of maximal aerobic ATP synthesis, as proposed herein, will be highly correlated with lipid oxidation capacity. Furthermore, we believe that improvements in lipid oxidation [and coupling of beta oxidation to complete oxidation as* CO_2*] are more important than improvements in mitochondrial mass per se. This is consistent with a large body of literature demonstrating that lipid intermediates are increased in T2DM [e.g. long-chain CoAs, acyl carnitines, ceramides, DAGs, etc.] and supports our hypothesis that lipid oxidation will be improved during exercise training in T2DM. Importantly, this proposal will test both of these possibilities.*

B6. Aerobic Training and HbA1c: Although there is strong evidence that aerobic exercise provides substantial health benefits in individuals with T2DM, the data to support improvements in HbA1c following aerobic exercise training are surprisingly weak. There is conflicting evidence on the effect of aerobic training on HbA1c. In a meta-analysis by Boule et al. (55) consisting of 12 aerobic training studies and 2 resistance training studies, exercise reduced HbA1c by 0.66%. However, many of the included studies had significant weaknesses such as a lack of randomized groups, small sample size (exercise groups n<20) and short study duration. Because HbA1c is a measure of average blood glucose concentration over an 8- to12-week period, studies where the duration of training was13 weeks or less (8 of 11 studies) may underestimate the effect of aerobic training on the concentration of HbA1c. Only one study had a trial period longer than 18 weeks. In summary, though the data are limited to a number of small studies, it is widely accepted that aerobic training may improve HbA1c concentration in individuals with T2DM.

B7. Resistance training and mitochondrial biogenesis/fat oxidation: In general, resistance training increases muscle cross-sectional area (+8 to 10%) (56); whereas mitochondrial volume, after correction for changes in muscle cross-sectional area, does not change (57). One notable exception to this is the remarkable increase in mitochondrial function measured by in vivo ^{31}P magnetic resonance spectroscopy (56) in resistance trained elderly volunteers. See preliminary data section C.

B8. HbA1c and Resistance Training: The available studies pertaining to resistance training and HbA1c conflict and are deficient in number and quality. Two studies report no improvement in HbA1c with resistance training (58, 59). However, these studies had small samples sizes (9 exercisers) and were of short duration (5-10 weeks). Two longer studies (24 and 16 weeks, respectively) reported decreases of 1.2% and 1.1% in HbA1c, respectively. Unfortunately, due to the methodological issues these two studies provide little help in quantifying the independent effect of resistance training on HbA1c. For example, Dunstan et al. (60) included weight loss in the intervention, and in a study by Castaneda et al. (61) the resistance training group greatly increased their daily physical activity outside the study, which consisted almost entirely of aerobic activity, while the control group did not. Taken as a whole, these studies suggest resistance training may improve HbA1c and mitochondrial function in individuals with T2DM. However, there is a need for an adequately powered, well-controlled study of long duration examining the effect of resistance training alone on mitochondrial function and fat oxidation in individuals with T2DM. *To our knowledge, there is no data on the effect of resistance training to increase mitochondrial biogenesis in T2DM [as it does in the elderly]. Thus, this aim of the study is novel, innovative and will fill a void in our understanding of how resistance training may improve mitochondrial function and fat oxidation in T2DM.*

B9. HbA1c and Combination Training: In September 2007, a report was published that compared the changes in HbA1c concentration in combined aerobic and resistance training to either of these training modes alone. Sigal et al. studied 251 patients with T2DM for six months who were randomized into one

of four groups: (1) aerobic training, (2) resistance training, (3) combination of aerobic and resistance training or (4) control. The absolute change in the HbA1c value in the aerobic exercise training group compared with the control group was -0.51% and -0.38% in the resistance training group. Combined exercise training resulted in an additional change in the HbA1c value of -0.46% compared with aerobic training alone and -0.59% compared with resistance training alone. One major limitation of this study, however, is that the total duration of exercise was greater in the combined exercise training group than in the aerobic and resistance training alone groups; thus, it is not clear if it is the combined exercise or simply the duration that leads to the greater decrease in HbA1c levels in the combined exercise training group. While this study supports the hypothesis of HART-D that aerobic and resistance training alone improve HbA1c levels in individuals with T2DM and the combined training is additive, it remains unclear what the effects of this training are on mitochondrial function and fat oxidation. The Castaneda et al. (61) study, however, is provocative. Though designed to be a resistance training only study, the resistance-training group increased their leisure time physical activity from 23 min per week to 120 min per week (>500%), while the control group decreased their activity (-44%, p=0.001 for between group differences). The decrease in HbA1c in the training group, -1.2%, is difficult to interpret due to the confounding effect of increased physical activity. However, this decrease supports the hypothesis that combined aerobic and resistance training may be more beneficial than either one alone on insulin sensitivity and mitochondrial function. *To our knowledge, there is no data addressing the hypothesis that resistance (or the combination aerobic and resistance training) increases mitochondrial biogenesis in T2DM. Thus, this aim of the study is novel, innovative and will fill a void in our understanding of how exercise training may improve mitochondrial function and fat oxidation in T2DM.*

C. Preliminary Studies

C1. Mitochondrial Mass in Young Men with a Family History of T2DM

Figure C1.A.

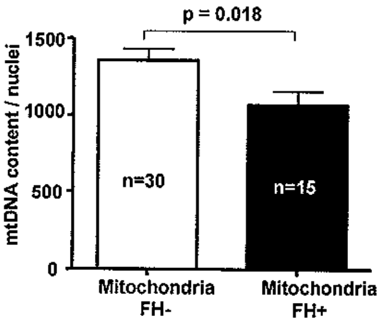

Key Result: establishes methods for the measurement of impaired mitochondrial biogenesis in individuals with a genetic susceptibility to T2DM

The ADAPT study is a short-term diet intervention study designed to examine inter-individual differences in whole body fat oxidation during the switch from iso-energetic standard to an iso-energetic high fat diet. Initial analyses have focused on the role of dietary fat on skeletal muscle gene expression (16). In an analysis of 50 healthy young men in this cohort, we tested the hypothesis that the capacity for mitochondrial mass on a high fat diet (HFD) was influenced in young FH + men. As shown in Figure C1.A., we found a reduction in muscle mitochondrial content but not insulin sensitivity, body fat or visceral fat discriminated between subjects with and without a family history of diabetes.

C2. Defects In Fat Oxidation Are Present In Ex Vivo Cultures Of Skeletal Muscle From Inflexible Donors

Key Result: establishes the techniques for the measurement of fat oxidation (^{13}C palmitate) *ex vivo* at PBRC and a role for impaired fat oxidation as an intrinsic defect in individuals with a genetic predisposition to T2DM.

The principal finding from this analysis of the ADAPT study is that metabolic switching, represented by dynamic changes in fatty acid oxidation in muscle cells cultured *ex vivo*, reflects the metabolic characteristics of the cells' donor. Adaptability, the capacity of myotubes to increase fat oxidation with a high palmitate concentration, was positively correlated with metabolic flexibility, insulin sensitivity and aerobic capacity and inversely associated with body fat and fasting insulin levels (63). Since its inception in May 2007, HART-D has randomized 64 participants and MARTi has completed 14 baseline measurements. *Ex vivo* fat oxidation of multiple substrates (palmitate, octonoate, pyruvate, acetate) in the skeletal muscle tissue of 10 MARTi II baseline completers has been measured and are presented in Figure C2 using 2 sedentary non-obese healthy individuals from ACTIV as controls.

Figure C2.

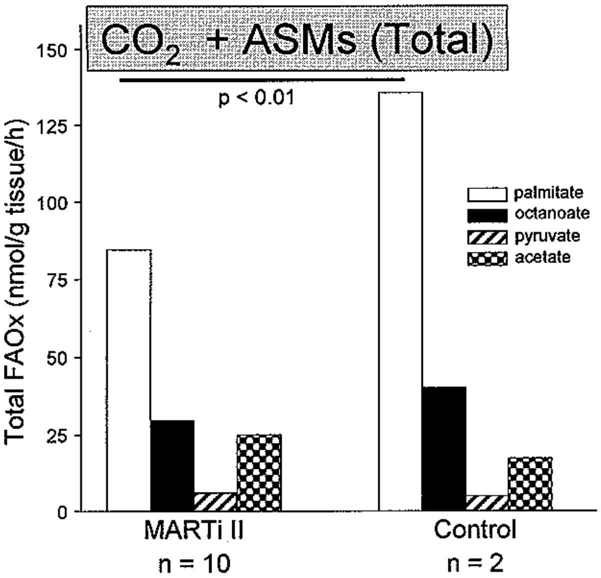

C3. Summary of the previously funded HART-D Protocol (Attached As Appendix)

Key Result: A substantial amount of prior work informs the design and conduct of a 9-month exercise intervention in T2DM. Health Benefits of Aerobic and Resistance Training in individuals with type 2 diabetes (HART-D). HART-D was reviewed by the PRDP review group in February 2006 (priority score = 141, percentile 4.9), and was funded in July 2006. The start of the study was delayed as Dr. Church assumed his new position at PBRC and as the infrastructure was set up in the PBRC clinical structure. The study began in May 2007, and currently has 64 randomized participants. In HART-D, 360 sedentary women and men with T2DM will be randomly assigned to an aerobic exercise training only group (AT), a resistance training only group (RT), a combination of aerobic plus resistance training (AT+RT) or a stretching and relaxation group (SR) to serve as a non-exercise control. The primary outcome of the parent trial is hemoglobin A1c (HbA1c). The AT individuals will participate in 3 or 4 training sessions each week for 9 months progressing to a total energy expenditure of 12 kcal/kg/week (KKW), which is an exercise dose consistent with the current public health recommendations for physical activity for individuals with T2DM. The target exercise intensity will be 50%-80% of baseline VO_2max. The RT group will participate in 3 sessions per week (30-45 minutes each), which focuses on large muscle groups at an intensity of 40-80% of 1-repetition maximum for the given exercise. Individuals in the AT+RT group will receive 10 KKW of aerobic training and a reduced resistance-training regimen of 2 sessions per week at 20 minutes per session to match duration of exercise across all 3 groups. This combination regimen represents the exact exercise recommendations of the American College of Sports Medicine (ACSM) and the American Diabetes Association (ADA). (7, 8) Simply stated, HART-D will examine if a hybrid program of AT+RT is superior to AT alone (or RT alone) in reducing HbA1c and if a RT has comparable benefits in lowering HbA1c to a standard AT program. Details of the experimental design [i.e. the full protocol] are included in the appendix.

C4. Rationale

A large body of literature demonstrates that defects in mitochondrial mass and function [lipid oxidation] are present in T2DM. Aerobic exercise increases mitochondrial mass and function in T2DM and is associated with improvements in glycemic control [and probably mitochondrial function (two studies)]. Much less is known about the effects of resistance training on mitochondrial mass and function [lipid oxidation] and no data is available on the use of resistance training in T2DM. Given that defects in lipid oxidation and lipotoxicity have been demonstrated in T2DM but lack mechanisms to explain these defects, the conduct of this study will address an important void in the clinical literature. This study leverages the clinical trial structure of the HART-D parent protocol and, using state-of-the-art measures of mitochondrial content and function, tests a key hypothesis of the diabetes literature, specifically that

1. Mitochondrial function and fat oxidation will increase with both aerobic and resistance training relative to the control group and are additive when combined.
2. Critical components of the fat oxidation system improve with exercise training to 'couple' lipid uptake with *complete* mitochondrial oxidation.

Several other possible questions could be answered within the conduct of this proposal and several of these are mentioned within the methods. In contrast, this proposal focuses on *in vivo* and *ex vivo* measures of mitochondrial mass and function pre and post aerobic, resistance or the combination of the two *independent of diet induced changes in body mass.* This is important because diet /weight loss itself is hypothesized to have salutary effects on muscle function. The proposed studies do not include dietary restriction and data from previous exercise intervention studies (HART-D) do not show a change in body weight.

A large body of literature supports the hypotheses in this application. Specifically, there is a growing body of literature that demonstrates that defects in mitochondrial capacity and function are present in T2DM and are reversed by aerobic training (54, 64). However, there are several unanswered questions including a) the potential role of resistance training to reverse mitochondrial dysfunction in T2DM [see (56) and preliminary data] and b) how combined resistance and aerobic training might be additive in reversing mitochondrial dysfunction.

C5. Significance of the Research

This research will be the first test of the hypothesis that resistance training, and more importantly the combination of resistance and aerobic training, will reverse the established defects in mitochondrial function in T2DM. These studies build upon a growing body of literature in humans and pre-clinical models that supports the hypothesis that mitochondrial defects are fundamental in the pathogenesis of insulin resistance in T2DM. The combination of measuring mitochondrial function *ex vivo* and other state-of-the-art measures of mitochondrial mass and function (biopsy) is highly novel. We will provide definitive results on the effects of aerobic and resistance training on mitochondrial function in patients with T2DM.

D. Research Design and Methods

D1. Hypotheses to be Tested

1. Mitochondrial function and fat oxidation will increase with both aerobic and resistance training relative to the control group and are additive when combined.
2. Critical components of the fat oxidation system improve with exercise training to 'couple' lipid uptake with *complete* mitochondrial oxidation.

Specific Aim – to measure mitochondrial capacity and fat oxidation *(ex vivo studies)* in a subset of participants enrolled in HART-D.

We will determine the mechanism(s) by which exercise training improves lipid oxidation in T2DM by measuring key components of the lipid oxidation system in skeletal muscle *(ex vivo studies)*. Our hypothesis is that each of the following systems improves with exercise training and 'couples' lipid uptake to *complete* mitochondrial oxidation [hypotheses 5]:

1. serum adiponectin	[RIA]	*Sparks*
2. adiponectin receptors 1/2	[mRNA]	*Sparks*
3. PGC1α	[Western immunoblotting]	*Sparks / Gettys*
4. Mitochondrial mass	[mtDNA, citrate synthase and EM analysis]	*Sparks / Sellers*
5. ETC activity	[isolated mitochondria from skeletal muscle]	*Sparks*
6. Mitochondrial lipid oxidation	[homogenate of skeletal muscle]	*Sparks*
7. Topology of lipid droplets **[E]**	[association of lipid droplets with mitochondria - analysis of EM]	
	[lipid coat proteins by Western blotting]	*Moro / Burke*
8. mitochondrial lipid transport **[E]**	[Western immunoblotting ± confocal IHC]	*Sparks / Burke*

[E] = exploratory

D2. EXPERIMENTAL DESIGN

As explained earlier, these aims will be performed as an ancillary study to the planned HART-D study. The overall timeline is presented, below. With a 9m intervention, we anticipate that it will take 3y to complete final planning, recruiting, and measurements. The final batched laboratory analysis will occur in y4. This is consistent with the parent protocol. We will admit into this protocol the volunteers already screened in the HART-D study at the Pennington Biomedical Research Center.

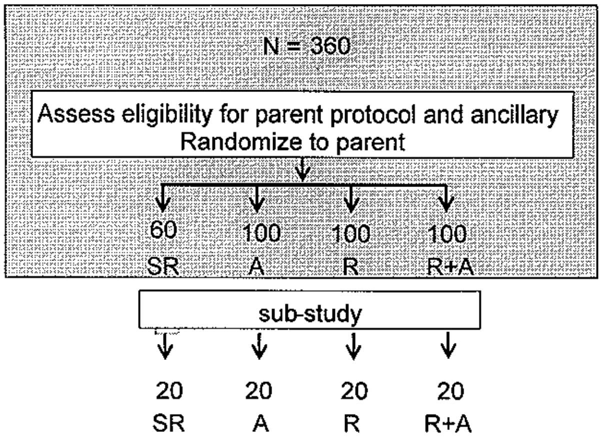

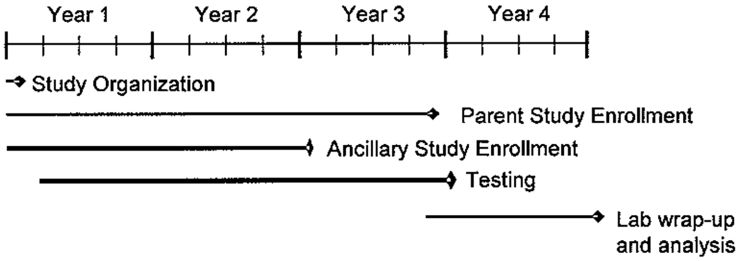

Recruiting is expected to take ~ 2y for the ancillary study. Eligible and willing volunteers will be allocated to treatment *and* participation in the ancillary. This will ensure balance of treatment within the ancillary protocol. Pennington HART-D participants will exercise (or not) in the parent / main protocol as planned (see abstract and appendix for details of the parent protocol).

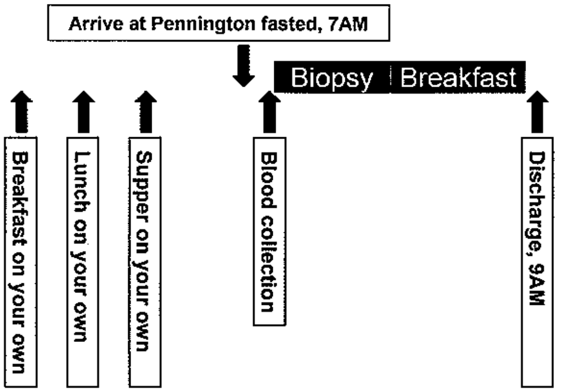

Volunteers will complete an 18 hour inpatient stay to perform the necessary studies at baseline and 9 months after beginning the exercise. They will start an ADA standard controlled diet (35%fat, 55%CHO, 15%PRO) the day before admittance to the PBRC Inpatient Unit. Participants will arrive to PBRC at 0700 fasted for at least 10 hours. At 0700, volunteers will proceed to the biopsy room where they will have fasting blood drawn for adiponectin and free fatty acids [72h post last exercise]. At 0730, volunteers will complete the skeletal muscle biopsy. Following the muscle biopsy, and ADA breakfast will be served and participants are discharged. The skeletal muscle biopsy of the vastus lateralis provides a mixed fiber type.

D3. STUDY POPULATION

Participants enrolled in the HART-D study will be required to meet the following inclusion/ exclusion criteria.

Inclusion

- Age 30 to 75 years.
- Type 2 diabetes determined by self-report with verification.
- 6.5% < HbA_{1c} <11%.
- Sedentary lifestyle: not being physically active ≥3 d/wk for 20 min each time for the previous 6 months, and not participating in regular resistance exercise.

Exclusion

- Inadequate control of co-morbid conditions.
- Resting blood pressure ≥160/100 mm Hg.
- Triglycerides ≥500 mg/dL.

- BMI > 45.
- Current use of an insulin pump or multiple insulin injections per day.
- Factors that may limit adherence to intervention or affect conduct of the trial
  - Unable or unwilling to communicate with staff, to provide written informed consent, or accept the randomized assignment.
  - Failure to complete behavioral run-in and baseline testing.
  - Hospitalization for depression in the last 6 months.
  - Not physically capable of performing the exercise required of the study protocols.
  - Consuming >14 alcoholic beverages per week.
  - Plans to be away >4 weeks in the next 9 months.
  - Lack of support from primary health care provider and/or family members.
  - Significant weight loss in the past year (>20 lbs) or current use of weight loss medications.
  - Current diagnosis of schizophrenia, other psychotic disorders, or bipolar disorder.
  - Another member of household is a participant or staff member of HART-D.
  - Bariatric surgery in the last 3 years.
  - Other temporary intervening event, such as sick spouse, bereavement, or recent move.
- Other medical, psychiatric, or behavioral limitations that, in the view of the principal investigator, may interfere with study participation or the ability to follow the intervention protocol.

Additional exclusion criteria are applied to the substudy to ensure safety for the skeletal muscle biopsy: family history of bleeding disorders, peripheral neuropathy.

D4. OUTCOME MEASURES

D4.1 Primary outcome

The primary endpoint of the study is the mechanisms related to changes in lipid oxidation *ex vivo* (biopsy).

D5. METHODS

D5.1 Muscle biopsy - A vastus lateralis muscle biopsy will be performed approximately 72h after the last exercise bout [since there is no exercise in the inpatient stay] using the technique of Bergstrom. *The leg (R vs. L) will be determined from a random number table and the second biopsy will be performed in the opposite leg to avoid artifacts of prior biopsy healing / scarring. This will be matched up to the leg that is used for the MRS studies.* After cleansing the skin with povidone-iodine solution, the skin, adipose tissue and skeletal muscle fascia are anesthetized using ≤ 5mL of a 50%/50% mixture of bupivicaine and lidocaine (final concentrations 1.0% and 0.125%). The skin is incised (0.75cm) with a #11 scalpel. The fascia fibers are separated with the blunt edge of the scalpel and the Bergstrom needle (4mm) inserted into the vastus lateralis. After suction is applied, approximately 50mg of tissue is cut and removed per pass a total of 5-6 passes will be performed. Pressure is applied and the skin is closed with sterile tape. After cleaning the sample, muscle will be snap frozen in liquid nitrogen or processed as described for EM, fatty acid oxidation, and mitochondrial isolation [for subsequent assays]. Dr. Smith has performed over one thousand Bergstrom biopsies.

A note on the tissue collections: The quantity of tissue samples has been carefully considered by the PI. The total amount of tissue needed for these assays is 260 mg. This amount of tissue is routinely collected at the Pennington Center (by SRS) with excellent anesthesia. For example in the recently completed CALERIE study at PBRC, there was very good acceptance with no refusals for second biopsies (n = 48).

assay	ETC, citrate synthase	fiber typing	fat oxidation	mRNA	electron microscopy	mtDNA	freeze-clamp and process
mg	50	25	75	30	20-30	10	50

D5.2 Overview of the skeletal muscle assays

See Fig B1 and B2 for a schematic of the fat oxidation / mitochondrial pathways. We have chosen to probe the structural / functional changes in the muscle lipid oxidation and the reported defects in mitochondrial electron chain activity. Obviously, there are many other parameters that could be measured such as signaling molecules like AMPK, allosteric regulators of lipid flux like malonylCoA and glycolytic pathways such as PDH/PDK4. Our focus is clearly based on our view of the literature and prior work. Additional studies could be performed on archival material (paraffin blocks, homogenized muscle, and frozen archived tissue) as the field advances. Similarly, our aim is not to measure specific toxic lipid species (ceramides, long chain coAs, etc) but as that field develops and broad-scope / ultra-sensitive assays become available (various metabolomic platforms for example) we may explore those areas as well. As such, we acknowledge that our experiment is biased and focused toward specific areas of mitochondrial metabolism that lie under the umbrella of our overall hypothesis that defects in mitochondrial function are present in T2DM and improvements in mitochondrial function and lipid oxidation occur with both aerobic and resistance training and are important determinants of the improvements in T2DM.

D5.3 Serum adiponectin – Serum adiponectin will be measured by RIA using the kit from Linco.

D5.4 Adiponectin receptors 1/2

[see Background and Preliminary Data for more details on the rationale for the measure of these genes]. Gene-specific primer and probe sets are provided below. The mRNA for these genes will be measured by qRT-PCR as previously described. Briefly, Trizol extraction will be followed by Qiagen RNA column and RNA quantified [and purity confirmed] by Agilent bioanalyzer (42, 71, 72).

Gene	Sense (5′- 3′)	Antisense (5′- 3′)	Probe (5′ FAM - BHQ1 3′)
ADIPOR1	ttcttcctcatggctgtgatgt	aagaagcgctcaggaattcg	tcactggagctggcctttatgctgc
ADIPOR2	atagggcagataggctggttga	ggatccgggcagcataca	tgatggccagcctctacatcacagga
PGC1a	tcctcttcaagatcctgctattac	gacggctgtagggcgatc	aagccactacagacaccgcacgc
PGC1b	cagccactcgaaggaacttca	cggatgcttggcgttctg	ctgaacacggccctctgctctcaca
CD36	agtcactgcgacatgattaatggt	ctgcaatacctggcttttctca	cagatgcagcctcatttccacctttg
RPLP0	ccattctatcatcaacgggtacaa	agcaagtgggaaggtgtaatcc	tctccacagacaaggccaggactcg

ADIPOR1, adiponectin receptor 1; ADIPOR2, adiponectin receptor 2; CD36, fatty acid transporter; PGC1a, peroxisome proliferative activated receptor gamma coactivator 1-a; peroxisome proliferative activated receptor gamma coactivator 1-b, and RPLP0, ribosomal protein large, P0 (our internal/loading control gene which we have found very stable in skeletal muscle biopsies).

D5.5 PGC1a

PGC1a protein in whole skeletal muscle will be measured by Western immunoblotting and normalized to β-actin.

D5.6 Mitochondrial mass

Mitochondrial mass will be measured on electron micrographs as previously described (42) and by the activity of citrate synthase and content of mtDNA. For the electron micrographs 0.5 ml fixative stock (6.25% Glutaraldehyde in 0.1 M Sodium Cacodylate Buffer, pH 7.4) is aliquoted into prelabeled 0.7 ml Eppendorf tubes. The sample [small (20-30 mg) without fat] is placed in the Eppendorf within seconds of the sample collection. The sample is stored at 4°C and shipped with a UN3373 Diagnostic specimen label on the outside and the morphometry of the EM images performed by Dr. Kevin Conley's group as previously described (73). For citrate synthase, an aliquot of the tissue homogenized for the measurement of ETC activity [homogenized in extraction buffer (0.1 M KH_2PO_4/Na_2PHO_4, 2 mM EDTA, pH = 7.2)] will be used for this assay. Citrate synthase (CS), activity will be determined in muscle homogenates using modifications of previously described methods (74). Briefly, CS activity will be measured at 37°C in 0.1 M Tris-HCl (pH 8.3) assay buffer containing 0.12 mM DTNB and 0.6 mM

oxaloacetate. Following an initial 2 min absorbance reading at 412 nm, the reaction will be initiated with the addition of 3.0 mM acetyl-CoA. The change in absorbance will be measured every 10 seconds for 7 min. Enzyme activity is expressed as μmole/mg protein/min. mtDNA content will be measured by quantitative real-time PCR as previously developed by us (6, 42, 71, 72, 75).

D5.7 Electron Transport Chain (ETC) activity

Analysis of mitochondrial ETC activity [NADH oxidase]. Mitochondria will be isolated using the methods of Rassmussen et al. (76) from 50 mg of vastus lateralis muscle, which has been cleaned of any visible adipose tissue. Citrate synthase activity will be assayed during the mitochondrial preparations to determine mitochondrial content of muscle, as well as percent recovery of mitochondria during the isolation procedures. Activity is assayed in the purified mitochondrial fraction as well as in the supernatant fractions generated during the isolation procedures, as described in Bezaire et al. (77). Our methods provide a mitochondrial recovery of approximately 30 - 40ug mitochondrial protein / 10mg muscle; we therefore do not anticipate any difficulties in completing the following functional assays of mitochondrial function. We have worked closely with Dr. MaryEllen Harper to learn this technique with excellent results. Activity of NADH:O_2 oxidoreductase will be determined in the total mitochondrial fraction by HPLC-based assay as described previously (3, 78). Briefly, mitochondrial preparations are suspended in medium containing 40 μg/ml alamethicin, 0.3mg/ml of 10mM Hepes, dioleoylphosphatidylcholine, 40mM KCl, 10mM histidine, 0.2 mM EGTA, 0.5 mg/ml BSA (pH 7.0 at 21°C) and dispensed as duplicate 50-μl aliquots (78). Samples are mixed with 0.5mM cytochrome c (electron acceptor between complex III and IV) and 7.5mM of NADH and incubated at 30°C for 10 min. The assay mixture is terminated by the addition of 1N H_2SO_4 and incubated at 30°C for 5 min to destroy the remaining NADH. Samples are neutralized by adding 50 U/ml of alcohol dehydrogenase for 60 min at 30°C. Blanks are mixed with H_2SO_4 before the addition of NADH and kept on ice. The samples are then mixed with methanol containing 2-amino-2-methyl-1-propanol and freezed clamped in liquid nitrogen to facilitate the aggregation of protein. The thawed samples are centrifuged at 5,000g for 5 min and the supernatant used for the HPLC analysis of NADH (3-5pmol detection limit) (3, 64, 78). Dr Moro is currently working with Dr Ravussin's laboratory to establish this method at PBRC.

D 5.8 Fiber typing - This data will provide important information about any changes in muscle composition, and will be essential for our interpretation of other findings. Immunohistochemical methods will be employed. Fiber type will be assessed using standard immunohistochemistry techniques, and triple chromophore staining of slides so that fibre types I, IIa IIb and IIx (the latter is unstained) are identified in single sections. Primary antibodies are from Developmental Studies Hybridoma Bank (University of Iowa) and used in conjunction with Vectastain ABC Alkaline Phosphatase kits (Vector, Burlington Ontario). Sections are mounted using water soluble antifade medium (VectaMount, Vector).

D5.9 Skeletal muscle lipid oxidation in muscle homogenates.

Assay will be performed as previously described (29). Briefly, muscle tissue will be minced and homogenized in a modified sucrose-EDTA medium (250 mM sucrose, 1 mM EDTA, and 10 mM Tris · HCl, pH 7.4). Fatty acid oxidation rates will be determined by measuring production of ^{14}C-labeled acid-soluble metabolites (ASM), a measure of tricarboxylic acid (TCA) cycle intermediates and acetyl esters (incomplete oxidation) (79), and [^{14}C]CO_2 (complete oxidation), by use of a custom made 24-well microtiter plate as previously described (63). Reactions will be initiated by adding 40 μl of whole homogenates to 160 μl of the incubation buffer (pH 7.4), yielding final concentrations (in mM) of 0.2 palmitate [vs. octanoate] ([1-^{14}C]palmitate or [1-^{14}C]octanoate at 0.5 μCi/ml), 100 sucrose, 10 Tris · HCl, 5 potassium phosphate, 80 potassium chloride, 1 magnesium chloride, 2 L-carnitine, 0.1 malate, 2 ATP, 0.05 CoA, 1 dithiothreitol, 0.2 EDTA, and 0.5% bovine serum albumin. After incubation for 60 min at 37°C, reactions will be terminated by adding 100 μl of 4 N sulfuric acid, and the CO_2 produced during the incubation will be trapped in 200 μl of 1 N sodium hydroxide that has been added to adjacent wells JCI (29). The acidified medium will be stored at 4°C overnight, and then ASM assayed in supernatants of the acid precipitate (79). Radioactivity of CO_2 and ASM will be determined by liquid scintillation counting by use of 4 ml of Uniscint BD (National Diagnostics). **The ratio of CO_2 to ASM will be analyzed by ANOVA, with time, treatment, and baseline value as factors in the model. An increase in the ratio**

of CO_2 to ASM will be interpreted as evidence of an increase in coupling of lipid β-oxidation to complete mitochondrial oxidation as CO_2. Similarly, the relative rates of oxidation of octanoate and palmitate will be compared pre-to-post exercise training using a similar model. An increase in the ratio of oxidation of palmitate to octanoate CO_2 will be interpreted as evidence of an increase in mitochondrial fatty acid transport and trigger the experiments on fatty acid transporters listed in D5.11.

D5.10 Topology of lipid droplets [E]

The number of lipid droplets 'connected' to mitochondria will be counted according to the method of Tarnopolsky, et al. Briefly, lipid droplets [200] will be counted and expressed as the % of lipid droplets that are touching a mitochondrial fragment (32). If this hypothesis is supported by the EM data,[i.e. that exercise increase the connections between lipid droplets and mitochondria in T2DM, then we will explore the expression (mRNA and protein) of the lipid coat proteins like ADRP which have been shown to increase in skeletal muscle with treatments that improve insulin sensitivity (33). An aliquot will be saved from the mitochondria isolation [required for the ETC activity assay]. Western immunoblotting will be performed using standard techniques and normalized to the content of citrate synthase. These measures will be interpreted in conjunction with the fatty acid oxidation assays in **D5.9**, above, and the whole body measures of fat oxidation (specifically metabolic chamber fat oxidation and exercise testing fat oxidation).

D5.11 Mitochondrial lipid transport proteins [E]

Recent reports from Spriet et al. demonstrate that CD36 is present in mitochondrial membranes and participates in the transport of long - chain fatty acids into the mitochondria (31). If there is an indication from the skeletal muscle fat oxidation assays that fatty acid transport is improved by exercise training (based on the a change in the relative oxidation rates for medium chain/ long-chain FA [octanoate/palmitate] pre-to-post training) then we will explore the hypothesis that CD36 [and possibly CPT-I] content in the mitochondria is increased by exercise training as one mechanism for increased mitochondrial fatty acid oxidation. An aliquot of mitochondria will be saved from the isolation [required for the ETC activity assay]. Western immunoblotting will be performed using standard techniques and normalized to the content of citrate synthase.

D 5.12 Data management / integration with the HART-D parent study.

The data collected in this work will be compiled in a database housed at the Pennington via data entry sheets (double entry) or direct electronic transfer from the instruments e.g. DEXA, metabolic carts, etc. The data will come from two sources: data from this work and data from the parent HART-D R01. Data from the PBRC database will be extracted, audited to ensure accuracy and placed into a SAS database for analysis. All analysis will be performed by the PI and co-Is with the aid of the biostatistician on the project.

D5.13 Study coordination / management and logistical considerations – Dr. Lauren M Sparks, as PI of this project, will coordinate the conduct of the study which will take place at several different laboratories (Smith, Conley, Ravussin, Church). The clinical protocol will be conducted at the Pennington Biomedical Research Center in the inpatient unit. The main clinical procedures not directly conducted by the HART-D research team occur under the organizational umbrella of the CNRU (blood draw for adiponectin and FFAs, muscle biopsy, etc.). Dr. Sparks takes responsibility for the day-to-day data collection procedures (muscle biopsy). Biopsy specimens will be collected under this protocol and transferred to the McIlhenny Skeletal Muscle Physiology Laboratory [Ravussin and Smith]. Fresh samples will be assayed as described by Dr. Sparks or a research associate (RA) in the laboratory, again under the organizational umbrella of the CNRU. This RA will be trained and supervised by Dr. Sparks. Some of the tissue samples obtained from the muscle biopsies will be sent to Dr. Kevin Conley at the University of Washington for further analysis.

References

1 McGarry JD. What if Minkowski had been ageusic? An alternative angle on diabetes. Science 1992; 258: 766-770.

2 Ravussin E, Smith SR. Increased fat intake, impaired fat oxidation, and failure of fat cell proliferation result in ectopic fat storage, insulin resistance, and type 2 diabetes mellitus. Ann N Y Acad Sci 2002; 967: 363-378.

3 Ritov VB, Menshikova EV, He J, Ferrell RE, Goodpaster BH, Kelley DE. Deficiency of subsarcolemmal mitochondria in obesity and type 2 diabetes. Diabetes 2005; 54: 8-14.

4 Simoneau JA, Colberg SR, Thaete FL, Kelley DE. Skeletal muscle glycolytic and oxidative enzyme capacities are determinants of insulin sensitivity and muscle composition in obese women. Faseb J 1995; 9: 273-278.

5 Petersen KF, Dufour S, Befroy D, Garcia R, Shulman GI. Impaired mitochondrial activity in the insulin-resistant offspring of patients with type 2 diabetes. N Engl J Med 2004; 350: 664-671.

6 Ukropcova B, Sereda O, de Jonge L, Bogacka I, Nguyen T, Xie H, et al. Family history of diabetes links impaired substrate switching and reduced mitochondrial content in skeletal muscle. Diabetes 2007; 56: 720-727.

7 Zinman B, Ruderman N, Campaigne BN, Devlin JT, Schneider SH. Physical activity/exercise and diabetes. Diabetes Care 2004; 27 Suppl 1: S58-62.

8 Albright A, Franz M, Hornsby G, Kriska A, Marrero D, Ullrich I, et al. American College of Sports Medicine position stand. Exercise and type 2 diabetes. Med Sci Sports Exerc 2000; 32: 1345-1360.

9 Simoneau JA, Kelley DE. Altered glycolytic and oxidative capacities of skeletal muscle contribute to insulin resistance in NIDDM. J Appl Physiol 1997; 83: 166-171.

10 Simoneau JA, Veerkamp JH, Turcotte LP, Kelley DE. Markers of capacity to utilize fatty acids in human skeletal muscle: relation to insulin resistance and obesity and effects of weight loss. Faseb J 1999; 13: 2051-2060.

11 Kelley DE, Mandarino LJ. Fuel selection in human skeletal muscle in insulin resistance: a reexamination. Diabetes 2000; 49: 677-683.

12 Smith SR, de Jonge L, Zachwieja JJ, Roy H, Nguyen T, Rood JC, et al. Fat and carbohydrate balances during adaptation to a high-fat. Am J Clin Nutr 2000; 71: 450-457.

13 Smith SR, de Jonge L, Zachwieja JJ, Roy H, Nguyen T, Rood J, et al. Concurrent physical activity increases fat oxidation during the shift to a high-fat diet. Am J Clin Nutr 2000; 72: 131-138.

14 Hansen KC, Zhang Z, Gomez T, Adams AK, Schoeller DA. Exercise increases the proportion of fat utilization during short-term consumption of a high-fat diet. Am J Clin Nutr 2007; 85: 109-116.

15 Bergouignan A, Schoeller DA, Normand S, Gauquelin-Koch G, Laville M, Shriver T, et al. Effect of Physical Inactivity on the Oxidation of Saturated and Monounsaturated Dietary Fatty Acids: Results of a Randomized Trial. PLoS Clin Trials 2006; 1: e27.

16 Sparks LM, Xie H, Koza RA, Mynatt R, Hulver MW, Bray GA, et al. A high-fat diet coordinately downregulates genes required for mitochondrial oxidative phosphorylation in skeletal muscle. Diabetes 2005; 54: 1926-1933.

17 Richardson DK, Kashyap S, Bajaj M, Cusi K, Mandarino SJ, Finlayson J, et al. Lipid infusion decreases the expression of nuclear encoded mitochondrial genes and increases the expression of extracellular matrix genes in human skeletal muscle. J Biol Chem 2005; 280: 10290-10297.

18 Watt MJ, Southgate RJ, Holmes AG, Febbraio MA. Suppression of plasma free fatty acids upregulates peroxisome proliferator-activated receptor (PPAR) alpha and delta and PPAR coactivator 1alpha in human skeletal muscle, but not lipid regulatory genes. J Mol Endocrinol 2004; 33: 533-544.

19 Bajaj M, Suraamornkul S, Kashyap S, Cusi K, Mandarino L, DeFronzo RA. Sustained Reduction in Plasma Free Fatty Acid Concentration Improves Insulin Action without Altering Plasma Adipocytokine Levels in Subjects with Strong Family History of Type 2 Diabetes. J Clin Endocrinol Metab 2004; 89: 4649-4655.

20 Bajaj M, Suraamornkul S, Romanelli A, Cline GW, Mandarino LJ, Shulman GI, et al. Effect of a sustained reduction in plasma free fatty acid concentration on intramuscular long-chain fatty Acyl-CoAs and insulin action in type 2 diabetic patients. Diabetes 2005; 54: 3148-3153.

21 Itani SI, Ruderman NB, Schmieder F, Boden G. Lipid-induced insulin resistance in human muscle is associated with changes in diacylglycerol, protein kinase C, and IkappaB-alpha. Diabetes 2002; 51: 2005-2011.

22 Pinnamaneni SK, Southgate RJ, Febbraio MA, Watt MJ. Stearoyl CoA desaturase 1 is elevated in obesity but protects against fatty acid-induced skeletal muscle insulin resistance in vitro. Diabetologia 2006; 49: 3027-3037.

23 Griffin ME, Marcucci MJ, Cline GW, Bell K, Barucci N, Lee D, et al. Free fatty acid-induced insulin resistance is associated with activation of protein kinase C theta and alterations in the insulin signaling cascade. Diabetes 1999; 48: 1270-1274.

24 Kim YB, Shulman GI, Kahn BB. Fatty acid infusion selectively impairs insulin action on Akt1 and PKClambda /zeta but not on glycogen synthase kinase-3. J Biol Chem 2002.

25 Goodpaster BH, Theriault R, Watkins SC, Kelley DE. Intramuscular lipid content is increased in obesity and decreased by weight loss. Metabolism 2000; 49: 467-472.

26 Ruderman NB, Saha AK, Vavvas D, Witters LA. Malonyl-CoA, fuel sensing, and insulin resistance. Am J Physiol 1999; 276: E1-E18.

27 Sahlin K, Mogensen M, Bagger M, Fernstrom M, Pedersen PK. The potential for mitochondrial fat oxidation in human skeletal muscle influences whole body fat oxidation during low-intensity exercise. Am J Physiol Endocrinol Metab 2007; 292: E223-230.

28 Kim JY, Koves TR, Yu GS, Gulick T, Cortright RN, Dohm GL, et al. Evidence of a malonyl-CoA-insensitive carnitine palmitoyltransferase I activity in red skeletal muscle. Am J Physiol Endocrinol Metab 2002; 282: E1014-1022.

29 Kim JY, Hickner RC, Cortright RL, Dohm GL, Houmard JA. Lipid oxidation is reduced in obese human skeletal muscle. Am J Physiol Endocrinol Metab 2000; 279: E1039-1044.

30 Zurlo F, Nemeth PM, Choksi RM, Sesodia S, Ravussin E. Whole-body energy metabolism and skeletal muscle biochemical characteristics. Metabolism 1994; 43: 481-486.

31 Bezaire V, Bruce CR, Heigenhauser GJ, Tandon NN, Glatz JF, Luiken JJ, et al. Identification of fatty acid translocase on human skeletal muscle mitochondrial membranes: essential role in fatty acid oxidation. Am J Physiol Endocrinol Metab 2006; 290: E509-515.

32 Tarnopolsky MA, Rennie CD, Robertshaw HA, Fedak-Tarnopolsky SN, Devries MC, Hamadeh MJ. The Influence of Endurance Exercise Training and Sex on Intramyocellular Lipid and Mitochondrial Ultrastructure, Substrate Use, and Mitochondrial Enzyme Activity. Am J Physiol Regul Integr Comp Physiol 2006.

33 Phillips SA, Choe CC, Ciaraldi TP, Greenberg AS, Kong AP, Baxi SC, et al. Adipocyte differentiation-related protein in human skeletal muscle: relationship to insulin sensitivity. Obes Res 2005; 13: 1321-1329.

34 He J, Goodpaster BH, Kelley DE. Effects of weight loss and physical activity on muscle lipid content and droplet size. Obes Res 2004; 12: 761-769.

35 Kelley DE, Simoneau JA. Impaired free fatty acid utilization by skeletal muscle in non-insulin-dependent diabetes mellitus. J Clin Invest 1994; 94: 2349-2356.

36 Petersen KF, Befroy D, Dufour S, Dziura J, Ariyan C, Rothman DL, et al. Mitochondrial dysfunction in the elderly: possible role in insulin resistance. Science 2003; 300: 1140-1142.

37 Kelley DE, He J, Menshikova EV, Ritov VB. Dysfunction of mitochondria in human skeletal muscle in type 2 diabetes. Diabetes 2002; 51: 2944-2950.

38 Amara CE, Shankland EG, Jubrias SA, Marcinek DJ, Kushmerick MJ, Conley KE. Mild mitochondrial uncoupling impacts cellular aging in human muscles in vivo. Proc Natl Acad Sci U S A 2007; 104: 1057-1062.

39 Lin J, Handschin C, Spiegelman BM. Metabolic control through the PGC-1 family of transcription coactivators. Cell Metab 2005; 1: 361-370.

40 Puigserver P, Spiegelman BM. Peroxisome proliferator-activated receptor-gamma coactivator 1 alpha (PGC-1 alpha): transcriptional coactivator and metabolic regulator. Endocr Rev 2003; 24: 78-90.

41 Koves TR, Li P, An J, Akimoto T, Slentz D, Ilkayeva O, et al. Peroxisome proliferator-activated receptor-gamma co-activator 1alpha-mediated metabolic remodeling of skeletal myocytes mimics exercise training and reverses lipid-induced mitochondrial inefficiency. J Biol Chem 2005; 280: 33588-33598.

42 Civitarese AE, Ukropcova B, Carling S, Hulver M, DeFronzo RA, Mandarino L, et al. Role of adiponectin in human skeletal muscle bioenergetics. Cell Metab 2006; 4: 75-87.

43 Lin J, Wu H, Tarr PT, Zhang CY, Wu Z, Boss O, et al. Transcriptional co-activator PGC-1 alpha drives the formation of slow-twitch muscle fibres. Nature 2002; 418: 797-801.

44 Cartoni R, Leger B, Hock MB, Praz M, Crettenand A, Pich S, et al. Mitofusins 1/2 and ERRalpha expression are increased in human skeletal muscle after physical exercise. J Physiol 2005; 567: 349-358.

45 Wright DC, Han DH, Garcia-Roves PM, Geiger PC, Jones TE, Holloszy JO. Exercise-induced mitochondrial biogenesis begins before the increase in muscle PGC-1alpha expression. J Biol Chem 2007; 282: 194-199.

46 Thyfault JP, Cree MG, Zheng D, Zwetsloot JJ, Tapscott EB, Koves TR, et al. Contraction of insulin resistant muscle normalizes insulin action in association with increased mitochondrial activity and fatty acid catabolism. Am J Physiol Cell Physiol 2006.

47 Long YC, Glund S, Garcia-Roves PM, Zierath JR. Calcineurin regulates skeletal muscle metabolism via coordinated changes in gene expression. J Biol Chem 2007; 282: 1607-1614.

48 Patti ME, Butte AJ, Crunkhorn S, Cusi K, Berria R, Kashyap S, et al. Coordinated reduction of genes of oxidative metabolism in humans with insulin resistance and diabetes: Potential role of PGC1 and NRF1. Proc Natl Acad Sci U S A 2003; 100: 8466-8471.

49 Mootha VK, Lindgren CM, Eriksson KF, Subramanian A, Sihag S, Lehar J, et al. PGC-1alpha-responsive genes involved in oxidative phosphorylation are coordinately downregulated in human diabetes. Nat Genet 2003; 34: 267-273.

50 Heilbronn LK, Smith SR, Ravussin E. The insulin-sensitizing role of the fat derived hormone adiponectin. Curr Pharm Des 2003; 9: 1411-1418.

51 Civitarese AE, Jenkinson CP, Richardson D, Bajaj M, Cusi K, Kashyap S, et al. Adiponectin receptors gene expression and insulin sensitivity in non-diabetic Mexican Americans with or without a family history of Type 2 diabetes. Diabetologia 2004; 47: 816-820.

52 Holloszy JO, Coyle EF. Adaptations of skeletal muscle to endurance exercise and their metabolic consequences. J Appl Physiol 1984; 56: 831-838.

53 Cortright RN, Sandhoff KM, Basilio JL, Berggren JR, Hickner RC, Hulver MW, et al. Skeletal muscle fat oxidation is increased in African-American and white women after 10 days of endurance exercise training. Obesity (Silver Spring) 2006; 14: 1201-1210.

54 Menshikova EV, Ritov VB, Toledo FG, Ferrell RE, Goodpaster BH, Kelley DE. Effects of weight loss and physical activity on skeletal muscle mitochondrial function in obesity. Am J Physiol Endocrinol Metab 2005; 288: E818-825.

55 Boule NG, Haddad E, Kenny GP, Wells GA, Sigal RJ. Effects of exercise on glycemic control and body mass in type 2 diabetes mellitus: a meta-analysis of controlled clinical trials. Jama 2001; 286: 1218-1227.

56 Jubrias SA, Esselman PC, Price LB, Cress ME, Conley KE. Large energetic adaptations of elderly muscle to resistance and endurance training. J Appl Physiol 2001; 90: 1663-1670.

57 MacDougall JD, Sale DG, Moroz JR, Elder GC, Sutton JR, Howald H. Mitochondrial volume density in human skeletal muscle following heavy resistance training. Med Sci Sports 1979; 11: 164-166.

58 Ishii T, Yamakita T, Sato T, Tanaka S, Fujii S. Resistance training improves insulin sensitivity in NIDDM subjects without altering maximal oxygen uptake. Diabetes Care 1998; 21: 1353-1355.

59 Baldi JC, Snowling N. Resistance training improves glycaemic control in obese type 2 diabetic men. Int J Sports Med 2003; 24: 419-423.

60 Dunstan DW, Daly RM, Owen N, Jolley D, De Courten M, Shaw J, et al. High-intensity resistance training improves glycemic control in older patients with type 2 diabetes. Diabetes Care 2002; 25: 1729-1736.

61 Castaneda C, Layne JE, Munoz-Orians L, Gordon PL, Walsmith J, Foldvari M, et al. A randomized controlled trial of resistance exercise training to improve glycemic control in older adults with type 2 diabetes. Diabetes Care 2002; 25: 2335-2341.

62 Blei ML, Conley KE, Kushmerick MJ. Separate measures of ATP utilization and recovery in human skeletal muscle. J Physiol 1993; 465: 203-222.

63 Ukropcova B, McNeil M, Sereda O, de Jonge L, Xie H, Bray GA, et al. Dynamic changes in fat oxidation in human primary myocytes mirror metabolic characteristics of the donor. J Clin Invest 2005; 115: 1934-1941.

64 Menshikova EV, Ritov VB, Fairfull L, Ferrell RE, Kelley DE, Goodpaster BH. Effects of exercise on mitochondrial content and function in aging human skeletal muscle. J Gerontol A Biol Sci Med Sci 2006; 61: 534-540.

65 Hoppeler H, Lindstedt SL. Malleability of skeletal muscle in overcoming limitations: structural elements. J Exp Biol 1985; 115: 355-364.

66 Conley KE, Jubrias SA, Esselman PC. Oxidative capacity and ageing in human muscle. J Physiol 2000; 526 Pt 1: 203-210.

67 Nguyen T, de Jonge L, Smith SR, Bray GA. Chamber for indirect calorimetry with accurate measurement and time discrimination of metabolic plateaus of over 20 min. Med Biol Eng Comput 2003; 41: 572-578.

68 de Jonge L, Nguyen T, Smith SR, Zachwieja JJ, Roy HJ, Bray GA. Prediction of energy expenditure in a whole body indirect calorimeter at both low and high levels of physical activity. Int J Obes Relat Metab Disord 2001; 25: 929-934.

69 Elia S, Livesey G. Energy Expenditure and fuel selection in biological systems: the theory and practice of calculations based on indirect calorimetry and tracer methods. In: Simopoulus AP (ed). Metabolic control of eating, energy expenditure and the bioenergetics of obesity World Review on Nutrition and Diet. Karger: Basel, 1992, pp 68-131.

70 Elia M, Livesey G. Theory and validity of indirect calorimetry during net lipid synthesis. Am J Clin Nutr 1988; 47: 591-607.

71 Bogacka I, Ukropcova B, McNeil M, Gimble JM, Smith SR. Structural and functional consequences of mitochondrial biogenesis in human adipocytes in vitro. J Clin Endocrinol Metab 2005; 90: 6650-6656.

72 Bogacka I, Xie H, Bray GA, Smith SR. Pioglitazone induces mitochondrial biogenesis in human subcutaneous adipose tissue in vivo. Diabetes 2005; 54: 1392-1399.

73 Hoppeler H, Kleinert E, Schlegel C, Claassen H, Howald H, Kayar SR, et al. Morphological adaptations of human skeletal muscle to chronic hypoxia. Int J Sports Med 1990; 11 Suppl 1: S3-9.

74 Heilbronn LK, Civitarese AE, Bogacka I, Smith SR, Hulver M, Ravussin E. Glucose tolerance and skeletal muscle gene expression in response to alternate day fasting. Obes Res 2005; 13: 574-581.

75 Civitarese AE, Carling S, Heilbronn LK, Hulver MH, Ukropcova B, Deutsch WA, et al. Calorie Restriction Increases Muscle Mitochondrial Biogenesis in Healthy Humans. PLoS Med 2007; 4: e76.

76 Rasmussen SK, Urhammer SA, Pizzuti A, Echwald SM, Ekstrom CT, Hansen L, et al. The K121Q variant of the human PC-1 gene is not associated with insulin resistance or type 2 diabetes among Danish Caucasians. Diabetes 2000; 49: 1608-1611.

77 Bezaire V, Hofmann W, Kramer JK, Kozak LP, Harper ME. Effects of fasting on muscle mitochondrial energetics and fatty acid metabolism in Ucp3(-/-) and wild-type mice. Am J Physiol Endocrinol Metab 2001; 281: E975-982.

78 Ritov VB, Menshikova EV, Kelley DE. High-performance liquid chromatography-based methods of enzymatic analysis: electron transport chain activity in mitochondria from human skeletal muscle. Anal Biochem 2004; 333: 27-38.

79 Veerkamp JH, Zevenbergen JL. Effect of dietary fat on total and peroxisomal fatty acid oxidation in rat tissues. Biochim Biophys Acta 1986; 878: 102-109.

80 Short KR, Vittone JL, Bigelow ML, Proctor DN, Rizza RA, Coenen-Schimke JM, et al. Impact of aerobic exercise training on age-related changes in insulin sensitivity and muscle oxidative capacity. Diabetes 2003; 52: 1888-1896.
